# Quantifying expression and metabolic activity of genes regulated by pregnane X receptor in primary human hepatocyte spheroids

**DOI:** 10.1371/journal.pcbi.1012886

**Published:** 2025-04-15

**Authors:** Lukáš Lochman, Ellen Tanaka Kahiya, Bechara Saade, Tomáš Smutný, Jurjen Duintjer Tebbens, Petr Pávek, Veronika Bernhauerová

**Affiliations:** 1 Department of Pharmaceutical Chemistry and Pharmaceutical Analysis, Faculty of Pharmacy in Hradec Králové, Charles University, Hradec Králové, Czech Republic; 2 Department of Biophysics and Physical Chemistry, Faculty of Pharmacy in Hradec Králové, Charles University, Hradec Králové, Czech Republic; 3 Department of Pharmacology and Toxicology, Faculty of Pharmacy in Hradec Králové, Charles University, Hradec Králové, Czech Republic; 4 Department of Artificial Intelligence, Institute of Computer Science of the Czech Academy of Sciences, Prague, Czech Republic; University of California, UNITED STATES OF AMERICA

## Abstract

Xenoreceptors of the nuclear receptor superfamily, such as pregnane X receptor (PXR), are liver-enriched ligand-activated transcription factors regarded as crucial sensors in xenobiotic exposure and detoxification. PXR controls transcription of many drug-handling genes and influx/eﬄux transporters, thus playing a crucial role in drug metabolism and excretion. Liver functions have been studied using primary human hepatocytes (PHHs), which, when conventionally cultured, undergo rapid de-differentiation, leaving them unsuitable for long-term studies. Recently, 3D PHHs called spheroids have emerged as an *in vitro* model that is similar to *in vivo* hepatocytes regarding phenotype and function and represents the first *in vitro* model to study the long-term regulation of drug-handling genes by PXR. In this study, we used mathematical modelling to analyze the long-term activation of PXR in 3D PHHs through expression kinetics of three key PXR-regulated drug-metabolizing enzymes, CYP3A4, CYP2C9, and CYP2B6 and the P-glycoprotein eﬄux transporter encoding gene, MDR1. PXR action in 3D PHHs was induced by the antibiotic rifampicin at two clinically relevant concentrations. The results confirmed that high rifampicin concentrations activated PXR nearly to its full capacity. The analysis indicated the highest PXR-induced transcription rate constant for CYP2B6. The rate constant dictating mRNA degradation associated with activated PXR was highest for CYP3A4. Moreover, we measured the metabolic activity of CYP3A4, CYP2C9, and CYP2B6 and quantified their metabolic rate constants. Metabolic activity rate constant of CYP3A4 was found to be the highest whereas that of CYP2B6 was found to be the lowest among the studied enzymes. Our results provide important insight into the regulation of PXR-target genes in 3D PHHs and show that mRNA expression and metabolic activity data can be combined with quantitative analysis to reveal the long-term action of PXR and its effects on drug-handling genes.

## Author summary

Pregnane X receptor (PXR) is a ligand-activated transcription factor which is essential for xenobiotic metabolism in the liver and intestine. Drug-handling genes, such as CYP3A4, CYP2C9, and CYP2B6 and the eﬄux transporters, such as P-glycoprotein encoded by the MDR1 gene, are controlled by PXR to facilitate metabolism and eﬄux of the majority of currently used drugs. The function of drug-handling genes has principally been studied in conventionally cultured 2D primary human hepatocytes (PHHs) which are not suitable for studying the long-term response to PXR activation due to early de-differentiation changes in the PHH-specific phenotype. There is a need to describe and quantify the processes involved in the long-term responses in the target gene regulation due to PXR activation to better understand drug metabolism regulation. The significance of our research is in quantifying the activation of PXR through pairing quantitative analysis and experimental data obtained from 3D PHH spheroids which allowed us to study the liver functions for long time periods. This work enhances our understanding of the responses to PXR activation at the level of mRNA expression and metabolic activity of drug-handling PXR-controlled genes.

## Introduction

Living organisms frequently encounter a variety of foreign substances, including drugs and toxins. To effectively identify and defend against these xenobiotics, the human body has developed a complex protective mechanism that is controlled by ligand-regulated nuclear receptors, such as the pregnane X receptor (PXR) [1–4]. PXR, primarily located in the liver, serves as a crucial transcription factor that acts as a xenosensor and responds to various chemical ligands through direct interactions [1,4–7]. Among these ligands, antibiotic rifampicin stands out in research studies as a model activator of PXR [5,8–10]. Rifampicin has been demonstrated to trigger substantial alterations in gene expression across the genome of primary human hepatocytes (PHHs), indicating the wide-ranging impact of PXR activation on liver gene expression patterns [8,11].

PXR controls drug-metabolizing cytochrome P450 (CYP) enzymes, in particular isoforms, such as CYP3A4, CYP2C9, and CYP2B6, as well as drug transporters, such as P-glycoprotein encoded by the multidrug resistance 1 (MDR1) gene. The expression of these genes is upregulated following the activation of PXR by rifampicin [9,12–14]. CYP3A4 is an important enzyme in the human that is responsible for metabolizing more than a half of drugs and xenobiotics, such as midazolam [15–17], which is mainly used for anesthesia and procedural sedation as well as utilized experimentally as a probe CYP3A4 substrate [18,19]. CYP2C9 and CYP2B6 also contribute significantly to drug metabolism. CYP2C9 substrates include nonsteroidal anti-inflammatory drugs (NSAIDs), for example, diclofenac [20–25]. CYP2B6 is the main enzyme that enables the metabolic conversion of bupropion, a drug used for treatment of depression, into its metabolite hydroxy bupropion [26–30]. Induced levels of CYP proteins thus affect the effectiveness of the administered drugs and even cause therapy failure. Additionally, MDR1 plays a central role in cellular eﬄux. High levels of MDR1 proteins increase the transport of compounds out of cells, such as hepatocytes [31–33], thus further affecting the treatment effectiveness. Therefore, understanding the pharmacologically-induced modulation of PXR activation might provide valuable insights into the role of PXR in detoxification processes and drug resistance [34]. Additionally, these genes play a key role in determining the bioavailability, clearance, and efficacy of drugs [1], emphasizing the importance of understanding their regulation to achieve optimal therapeutic outcomes.

Mathematical models are useful to understand and quantify the expression kinetics of PXR-regulated genes *in vitro* and *in vivo* (reviewed in [35]). However, the number of studies that utilize mechanistic mathematical models of PXR activation is rather limited and mostly focused on the regulation of the human CYP3A4 by rifampicin [36,37] and cortisol [38] in PHHs. Additionally, other theoretical models described the rodent PXR regulation of its target genes (CYP3A1/2) in rats with PXR ligands, namely the glucocorticoid drug dexamethasone [38,39] and the steroidal antiglucocorticoid drug pregnenolone-16*α*-carbonitrile [40]. Mathematical models that would accurately describe the expression kinetics of other PXR-regulated drug-handling genes, such as CYP2C9 and CYP2B6 enzymes or MDR1 transporter, or attempt to collectively relate the up-regulated expression kinetics of drug-handling genes to the activation of PXR are not available. Moreover, the metabolic activity of these PXR-regulated CYPs has been widely omitted in mathematical models and thus not properly quantified.

In this study, we combine a mechanistic mathematical model of PXR activation with data obtained from the experimental treatments of 3D primary human hepatocyte spheroids (3D PHHs) with PXR agonist rifampicin to identify how different rifampicin concentrations affect kinetic rates and collective control of PXR-regulated drug handling genes, CYP3A4, CYP2C9, CYP2B6, and MDR1. Unlike conventionally used 2D PHH monolayers, which de-differentiate within several hours after seeding [9,41], 3D PHHs exhibit high similarity in the expression of absorption, distribution, metabolism, and excretion protein patterns to those of the normal *in vivo* liver cells [9,41–43], which proposes 3D PHHs as a highly physiologically relevant *in vitro* model. More importantly, 3D PHHs have proven to maintain relatively stable hepatocyte-specific features for long time periods which has allowed us to fully observe the effects of PXR activation on the expression and metabolic activity of the drug-handling genes.

**Fig 1:**
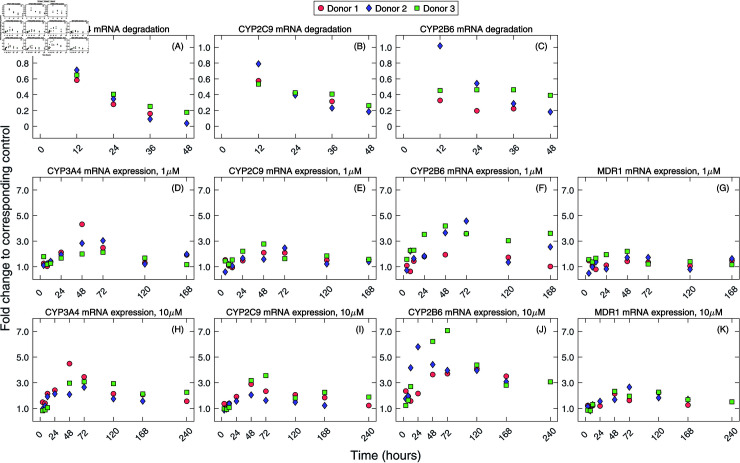
mRNA expression profiles of PXR-controlled drug-metabolizing enzymes and MDR1 transporter. **(A)–(C)** Fold changes of mRNA levels in 3D PHHs at indicated time points following SPA70 treatment (10 µM) were related to the mRNA level at 72 h of rifampicin pre-treatment (set as 1). **(D)–(K)** mRNA expression profiles in 3D PHHs after treatment with rifampicin at 1 µM ((D)–(G)) or 10 µM ((H)–(K)) concentrations for indicated time. Data are shown as the fold change expression relative to corresponding DMSO control.

Fitting the mathematical model to the gene expression and metabolic activity kinetic data allowed us to quantify the rates driving PXR activation in 3D PHHs and the PXR-induced transcription and degradation of CYP3A4, CYP2C9, CYP2B6, and MDR1, including the metabolic rates of CYP3A4, CYP2C9, and CYP2B6. The analysis confirmed that higher rifampicin concentrations lead to higher activation of PXR. We showed that CYP2B6 had the highest rate constant controlling the PXR-induced transcription rate whereas CYP3A4 had the highest rate constant driving the mRNA degradation rate. On the contrary, the rate constant controlling the metabolic rate of CYP3A4 was the highest among all CYPs. Metabolic activity of CYP3A4 was determined in two different experimental scenarios. In the first instance, 3D PHHs were treated with CYP3A4-specific probe substrate, midazolam, while in the second instance, 3D PHHs were treated with a mixture of CYP-specific probe substrates, midazolam (CYP3A4), diclofenac (CYP2C9), and bupropion (CYP2B6). The results showed that the rate constant associated with the metabolic rate of CYP3A4 was higher in the latter scenario. Together, these analysis highlight the underlying processes that drive PXR activation in 3D PHHs and the regulation of its drug-handling genes resulting from different treatment scenarios.

## Results

### Expression data of PXR-regulated genes

Data in panels A, B, and D–K in Fig 1 were previously published in [9]. Briefly, 3D PHHs gained from three donors were treated with the antibiotic rifampicin at the concentrations of 1 µM (low concentration) and 10 µM (high concentration). mRNA levels of CYP3A4, CYP2C9, CYP2B6, and MDR1 genes were measured for 168 h. In the case of high rifampicin concentration treatment, we newly measured mRNA levels at 240 h (Fig 1H–K). In separate experiments, degradation of CYP3A4, CYP2C9, and CYP2B6 mRNA levels were determined by inhibiting the PXR-pathway by exposing 3D PHHs to SPA70, a well-known PXR antagonist [44], at 72 h post treatment with 10 µM rifampicin. The resulting mRNA levels were analyzed at times 12 h, 24 h, 36 h, and 48 h and quantified (Fig 1A–1C). Contrary to [9], data in Fig 1 are presented with several modifications. Individual mRNA changes for each 3D PHH donor are displayed instead of the averages that were previously shown in [9]. Such data visualization revealed discrepancies in the peaks of CYP2B6 mRNA expression profiles at 1 µM and 10 µM rifampicin concentrations for donors 2 and 3 while for other genes, the mRNA expression profiles exhibited a higher degree of homogeneity. Specifically, CYP2B6 mRNA levels reached their maxima at 72 h and 48 h post treatment with 1 µM rifampicin for donors 2 and 3, respectively, while the reverse was observed for these donors following treatment with 10 µM rifampicin; CYP2B6 mRNA levels reached their maxima at 24 h and 72 h post treatment for donors 2 and 3, respectively. We also note that the degradation of CYP2B6 mRNA levels in 3D PHHs were not previously published (Fig 1C). Details regarding the experiments and data are provided in Materials and Methods and in [9].

**Fig 2:**
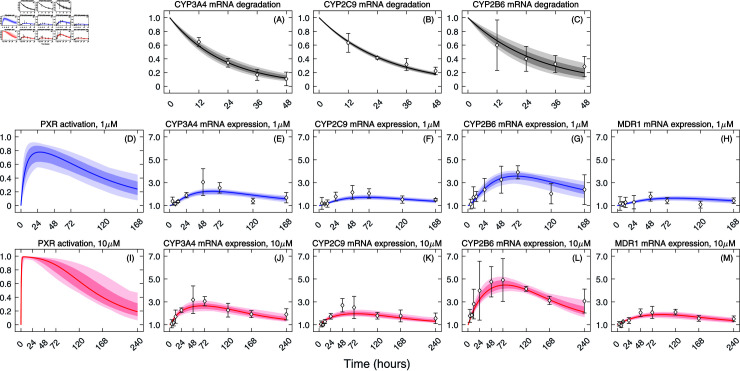
Fits of the gene expression model to mRNA expression data. Equations (6)–(10) and (23) were simultaneously fitted to **(A)–(C)** mRNA degradation data, mRNA expression data from 3D PHHs treated with **(D)–(H)** 1 µM (blue) and **(I)–(M)** 10 µM (red) of rifampicin using MCMC. Accepted parameter sets were used to generate solutions which are displayed as filled areas. Light filled area represents all MCMC-accepted solutions while dark filled area represents 95% credible bands. The solutions corresponding to the MCMC maximum likelihood estimate are displayed as a solid line. Data are displayed as mean  ±  standard deviation for three donors per time point and are expressed as the fold change expression relative to corresponding control.

### Quantitative description of PXR activation and expression of PXR-regulated genes

We quantified the impact of rifampicin on the activation of PXR by simultaneously fitting the gene expression model (Equations (6)–(10) and (23)) to gene expression kinetic data (Fig 2) using the MCMC routine (details of the fitting procedure are provided in Materials and methods). Fits are consistent (within errorbars) with the gene expression profiles of all PXR-regulated genes. The model predicted that PXR activation was more rapid and reached a higher peak when 3D PHHs were treated with the high concentration of rifampicin compared to the low concentration of rifampicin. For the low rifampicin concentration treatment, the peak activation of PXR associated with the best-fit parameters (Table 1) was achieved at 24.1 h post treatment at which point 77.7% of the total PXR was activated. For the high rifampicin concentration treatment, the peak activation of PXR was achieved at 5.8 h post treatment at which point 99.0% of the total PXR was activated. PXR was activated by rifampicin at the maximum rate constant $k_{\text{pxr,max}}$ estimated at 0.143 µM^−1^h^−1^. In the gene expression model (Equations (6)–(10) and (23)), we assumed that the ability of PXR to become activated and thus engage in transcription of CYPs and MDR1 diminishes over time, for example, due to depletion of transcriptional co-factors, despite that rifampicin concentration remains at high levels over long time periods. We modeled this decreasing ability of PXR to become activated by rifampicin by reducing $k_{\text{pxr,max}}$ by the factor $e^{-k_{\text{r}}\;t}$, where $k_{\text{r}}$ is the rate constant and was estimated at 0.049 h^−1^. Activated PXR degraded at the rate constant $k_{{\text{pxr}}_{\text{deg}}}$ estimated at 0.011 h^−1^. We clarify that the rate constants $k_{\text{r}}$ and $k_{{\text{pxr}}_{\text{deg}}}$ describe two mechanistically distinct processes. While $k_{\text{r}}$ reduces the rate at which rifampicin-activated PXR is formed, $k_{{\text{pxr}}_{\text{deg}}}$ acts on already existing activated PXR and drives its degradation.

**Fig 3:**
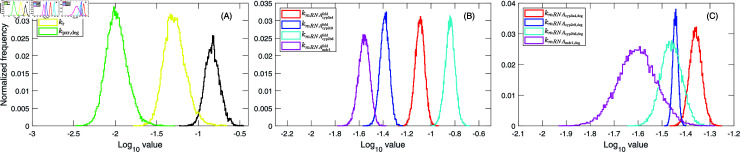
PXR- and gene-specific parameters. Parameter posterior histograms resulting from fitting the gene expression model (Equations (6)–(10) and (23)) to mRNA expression data from 3D PHHs treated with 1 µM and 10 µM of rifampicin. Parameters shown are **(A)** the maximum PXR activation rate constant, $k_{\text{pxr,max}}$, the rate constant controling reduction in PXR activation rate, $k_{\text{r}}$, and the activated PXR degradation rate constant, $k_{{\text{pxr}}_{\text{deg}}}$, **(B)** fold-transcription rate constants of CYP3A4, $k_{{mRNA}_{\text{cyp3a4}}^{\text{fold}}}$, CYP2C9, $k_{{mRNA}_{\text{cyp2c9}}^{\text{fold}}}$, CYP2B6, $k_{{mRNA}_{\text{cyp2b6}}^{\text{fold}}}$, and MDR1, $k_{{mRNA}_{\text{mdr1}}^{\text{fold}}}$, **(C)** mRNA degradation rate constants of CYP3A4, $k_{{mRNA}_{\text{cyp3a4,deg}}}$, CYP2C9, $k_{{mRNA}_{\text{cyp2c9,deg}}}$, CYP2B6, $k_{{mRNA}_{\text{cyp2b6,deg}}}$, and MDR1, $k_{{mRNA}_{\text{mdr1,deg}}}$. Additional histograms and pair-wise parameter correlation plots are in Fig B in S1 Text.

The PXR-dependent fold transcription rate constants differed between the PXR-regulated genes. CYP2B6 had the highest value of the PXR-dependent fold transcription rate constant, $k_{{mRNA}_{\text{cyp2b6}}}^{\text{fold}}$ (0.139 h^−1^), followed by that of CYP3A4, $k_{{mRNA}_{\text{cyp3a4}}}^{\text{fold}}$ (0.083 h^−1^), CYP2C9, $k_{{mRNA}_{\text{cyp2c9}}}^{\text{fold}}$ (0.040 h^−1^), and MDR1, $k_{{mRNA}_{\text{mdr1}}}^{\text{fold}}$ (0.026 h^−1^). The mRNA degradation rate constant was highest for CYP3A4, $k_{{mRNA}_{\text{cyp3a4,deg}}}$ (0.044 h^−1^), followed by that of CYP2C9, $k_{{mRNA}_{\text{cyp2c9,deg}}}$ (0.036 h^−1^), CYP2B6, $k_{{mRNA}_{\text{cyp2b6,deg}}}$ (0.034 h^−1^), and MDR1, $k_{{mRNA}_{\text{mdr1,deg}}}$ (0.023 h^−1^). The differences between all the PXR-dependent fold transcription and degradation rate constants were statistically significant (p $<10^{-5}$, Wilcoxon rank-sum test, details are in Materials and Methods). We note that MDR1 mRNA degradation rate constant, $k_{{mRNA}_{\text{mdr1,deg}}}$, was estimated directly from the long-term mRNA expression data of MDR1 (Fig 2H and 2M) because inhibiting PXR by its antagonist SPA70 did not result in a spontaneous decrease in MDR1 transcription (Fig A in S1 Text). Consequently, we could not exclude the contribution of the basal transcription of MDR1, which appears not to be exclusively maintained by PXR, to the degradation of MDR1 mRNA following PXR activation. In contrast, in [9] we demonstrated that SPA70-induced inhibition of PXR in DMSO control 3D PHHs resulted in a significant reduction of the basal transcription of CYP3A4 and CYP2C9 and the rate constants driving the mRNA degradation of these CYPs were comparable to the rate constants estimated for rifampicin-pretreated 3D PHHs in which SPA70 was applied at 72 h post rifampicin treatment. Therefore, direct comparison of the MDR1 and CYP mRNA degradation rate constants might not be straightforward. For instant visualisation, the posterior histograms of the PXR- and CYP-associated parameters are depicted in Fig 3. Visualizations of the relationships and correlations between individual parameters are depicted as pair-wise plots in Fig B in S1 Text. The best-fit values and 95% credible intervals of all parameters are listed in Table 1.

**Table 1:** Parameter estimates for gene expression model. Best-fit parameter values and 95% credible intervals (CIs) obtained from fitting the gene expression model (Equations (6)–(10) and (23)) to gene expression and degradation data.

Parameter	Value [95% CI]
maximum PXR activation rate constant, $k_{\text{pxr,max}}$ (µM^−1^h^−1^)	0.143 [0.107; 0.208]
rate constant controling reduction in PXR activation rate, $k_{\text{r}}$ (h^−1^)	0.049 [0.033; 0.088]
activated PXR degradation rate constant, $k_{{\text{pxr}}_{\text{deg}}}$ (h^−1^)	0.011 [0.007; 0.017]
PXR-dependent CYP3A4 mRNA fold transcription rate constant, $k_{{mRNA}_{\text{cyp3a4}}}^{\text{fold}}$ (h^−1^)	0.083 [0.069; 0.093]
CYP3A4 mRNA degradation rate constant, $k_{{mRNA}_{\text{cyp3a4,deg}}}$ (h^−1^)	0.044 [0.040; 0.048]
PXR-dependent CYP2C9 mRNA fold transcription rate constant, $k_{{mRNA}_{\text{cyp2c9}}}^{\text{fold}}$ (h^−1^)	0.040 [0.035; 0.047]
CYP2C9 mRNA degradation rate constant, $k_{{mRNA}_{\text{cyp2c9,deg}}}$ (h^−1^)	0.036 [0.034; 0.037]
PXR-dependent CYP2B6 mRNA fold transcription rate constant, $k_{{mRNA}_{\text{cyp2b6}}}^{\text{fold}}$ (h^−1^)	0.139 [0.123; 0.168]
CYP2B6 mRNA degradation rate constant, $k_{mRNA_{\text{cyp2b6,deg}}}$ (h^−1^)	0.034 [0.029; 0.041]
PXR-dependent MDR1 mRNA fold transcription rate constant, $k_{{mRNA}_{\text{mdr1}}}^{\text{fold}}$ (h^−1^)	0.026 [0.023; 0.033]
MDR1 mRNA degradation rate constant, $k_{{mRNA}_{\text{mdr1,deg}}}$ (h^−1^)	0.023 [0.018; 0.034]

### Metabolic activity data of PXR-regulated genes

3D PHHs were treated with rifampicin at the concentration of 10 µM for 24 h, 48 h, 72 h, and 120 h. At the indicated times, rifampicin-enriched medium was extracted and replaced with medium containing the CYP-specific probe substrate. Quantification of the CYP-specific metabolite was performed at 4 h following the treatment with the CYP-specific probe substrate (details are in Materials and Methods). We note that all CYPs exhibited activities higher than their baseline following PXR activation (Fig C in S1 Text).

**Fig 4:**
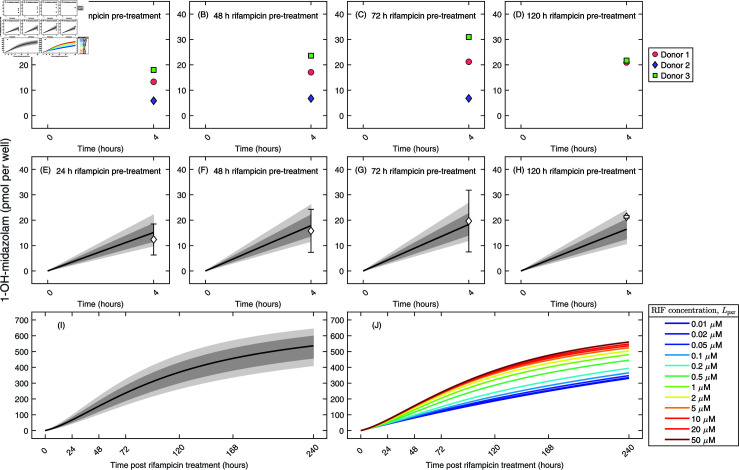
CYP3A4 activity in 3D PHHs. **(A)–(D)** 3D PHHs were treated with rifampicin (10 µM) for (A) 24 h, (B) 48 h, (C) 72 h, and (D) 120 h. Then, CYP3A4 activity was evaluated as the formation of 1-OH-midazolam following treatment with midazolam (10 µM) for 4 h. Data were collected from three PHH donors and presented in pmol per well. **(E)–(H)** Simultaneous fits of Equation (18) evaluated at CYP3A4 fold mRNA levels at times 24 h, 48 h, 72 h, and 120 h rifampicin post treatment to 1-OH-midazolam measurements using MCMC. Light filled area represents all MCMC-accepted solutions while dark filled area represents 95% credible bands. The CYP3A4 metabolic rate constant $k_{{\text{met}}_{\text{cyp3a4,ss}}}^{\text{fold}}$ was assumed to be a common parameter in the fitting. Data are displayed as mean  ±  standard deviation for three donors per time point and are expressed in pmol per well. **(I)** Predicted accumulation of 1-OH-midazolam was generated by solving the gene expression (Equations (6)–(10)) and metabolic activity (Equations (13)–(14)) models simultaneously using the best-fit parameter values from Tables 1 and 2. The solution corresponding to the MCMC maximum likelihood estimate is displayed as a solid line. Predicted accumulation of 1-OH-midazolam corresponding to the MCMC-accepted values of the parameter $k_{{\text{met}}_{\text{cyp3a4,ss}}}^{\text{fold}}$ are displayed as the filled area. Light filled area represents all MCMC-accepted solutions while dark filled area represents 95% credible bands. **(J)** Predicted CYP3A4 activities for different rifampicin concentrations, $L_{\text{pxr}}$, were generated using the best-fit parameter values from Tables 1 and 2. The initial substrate concentration, $S_{\text{cyp3a4},0}$, was 800 pmol per well.

Metabolic activities of CYP3A4, CYP2C9, and CYP2B6 were measured as the formation (expressed in pmol per well) of 1-hydroxy midazolam (1-OH-midazolam), 4-hydroxy diclofenac (4-OH-diclofenac), and hydroxy bupropion (OH-bupropion) following treatment with the mixture of the CYP3A4-specific probe substrate, midazolam, at the dose concentration of 10 µM (Fig D in S1 Text (panels A–D)), the CYP2C9-specific probe substrate, diclofenac, at the concentration of 10 µM (Fig E in S1 Text (panels A–D)), and the CYP2B6-specific probe substrate, bupropion, at the concentration of 40 µM (Fig F in S1 Text (panels A–D)) (herein referred to as the substrate mixture (sm)). To examine whether the metabolic activity of CYP3A4, which is the most important metabolizing enzyme, is influenced by metabolism of other substrates, we also measured the metabolic activity of CYP3A4 following treatment with midazolam only (herein referred to as the single substrate (ss)), at the concentration of 10 µM (Fig 4A–4D).

### Quantitative description of metabolic activity of PXR-regulated genes

We proposed to describe the CYP metabolic activity by the metabolic activity model (Equations (11)–(12)) which assumed that the CYP-specific substrate, $\left[S_{\text{cyp}}\right]$, was transformed into the metabolite, $\left[M_{\text{cyp}}\right]$, proportionally to the CYP mRNA expression levels, [$mRNA_{\text{cyp}}]$, and the reaction was controlled by the CYP-specific metabolic rate constant, $k_{{\text{met}}_{\text{cyp}}}$. We then transformed the metabolic activity model to relate the formation of the metabolite to the fold CYP mRNA expression levels, $\left[mRNA_{\text{cyp}}^{\text{fold}}\right]$, in which the reaction was newly controlled by the rate constant $k_{{\text{met}}_{\text{cyp}}}^{\text{fold}}$ (Equations (13)–(14)). To quantify the CYP-specific metabolic rate constants, we first evaluated the fold CYP mRNA expression levels from the gene expression model (Equations (7)–(9)) at 24 h, 48 h, 72 h, and 120 h using the best-fit parameter estimates (Table 1). Then, the solutions of the metabolic activity model (Equation (18)) evaluated at the corresponding fold CYP mRNA expression levels were simultaneously fitted to the CYP-specific metabolite measurements at the indicated times. We stress that the metabolic activity model was simultaneously fitted to the CYP-specific metabolite levels at all indicated times and the CYP-specific metabolic activity rate constant, $k_{{\text{met}}_{\text{cyp}}}^{\text{fold}}$, was set as a common parameter in the fitting process. The differences in the CYP-specific metabolite levels at the indicated times were thus assumed to be driven exclusively by varying CYP fold mRNA levels. Fits of the metabolic activity model to CYP3A4, CYP2C9, and CYP2B6 activity data are in Fig D in S1 Text (panels E–H), Fig E in S1 Text (panels E–H), and Fig F in S1 Text (panels E–H), respectively.

The rate constant at which CYP3A4 metabolizes midazolam into 1-OH-midazolam in the single substrate treatment scenario, $k_{{\text{met}}_{\text{cyp3a4,ss}}}^{\text{fold}}$, was estimated to be 2.18 × 10^−3^ h^−1^. Fits of the metabolic activity model to CYP3A4 activity data are in Fig 4E–H. In the substrate mixture treatment scenario, the rate constants at which CYP3A4, CYP2C9, and CYP2B6 metabolize midazolam into 1-OH-midazolam, $k_{{\text{met}}_{\text{cyp3a4,sm}}}^{\text{fold}}$, diclofenac into 4-OH-diclofenac, $k_{{\text{met}}_{\text{cyp2c9,sm}}}^{\text{fold}}$, and bupropion into OH-bupropion, $k_{{\text{met}}_{\text{cyp2b6,sm}}}^{\text{fold}}$, respectively, were determined to be 3.02 × 10^−3^ h^−1^, 1.41 × 10^−3^ h^−1^, and 0.85 × 10^−4^ h^−1^, respectively. These values indicate that CYP3A4 is the most efficient of all CYPs whereas CYP2B6 is the least efficient of all CYPs in metabolizing their respective substrates. The parameter posterior histograms of all CYP-specific metabolic rate constants are in Fig G in S1 Text. The best-fit values and 95% credible intervals of the CYP-specific metabolic rate constants are listed in Table 2.

Metabolic activity is usually measured only for very short time periods (ranging from several dozens of minutes to a few hours), because in these short windows the reaction is approximately linear which makes the back-calculation of the CYP metabolic rate straightforward [45]. However, these simple calculations are not able to explicitly account for the contribution of the varying CYP mRNA or protein levels to the overall metabolism. To consider varying levels of CYP mRNA, we predicted the long-term accumulation of the metabolites by combining the gene expression model (Equations (6)–(9)) using the best-fit parameter values in Table 1 together with the metabolic activity model (Equations (13)–(14)) using the estimated CYP metabolic rate constants in Table 2. Predicted levels of 1-OH-midazolam for the single substrate treatment scenario are depicted in Fig 4I. Predicted levels of 1-OH-midazolam, 4-OH-diclofenac, and OH-bupropion for the substrate mixture treatment scenario are in Fig D in S1 Text (panel I), Fig E in S1 Text (panel I), and Fig F in S1 Text (panel I), respectively. The highest levels of accumulated metabolite over the course of 240 h were associated with CYP3A4 mRNA expression and the lowest with CYP2B6 mRNA expression, despite that CYP2B6 mRNA expression was highest among the studied genes.

**Table 2:** Parameter estimates for metabolic activity model. Best-fit values of the CYP-specific metabolic rate constants and 95% credible intervals (CIs) obtained from fitting the solution (Equation (18)) of the metabolic activity model (Equations (15)–(16)) to the CYP-specific metabolic activity measurements. The CYP metabolic rate constants associated with the single substrate experiment are denoted with the subscript ‘ss’ and the CYP metabolic rate constants associated with the substrate mixture experiment are denoted with the subscript ‘sm’.

Parameter	Value [95% CI]
CYP3A4 metabolic rate constant, $k_{{\text{met}}_{\text{cyp3a4,ss}}}^{\text{fold}}$ (h^−1^)	2.18 [1.75; 2.18] × 10^−3^
CYP3A4 metabolic rate constant, $k_{{\text{met}}_{\text{cyp3a4,sm}}}^{\text{fold}}$ (h^−1^)	3.02 [2.76; 3.28] × 10^−3^
CYP2C9 metabolic rate constant, $k_{{\text{met}}_{\text{cyp2c9,sm}}}^{\text{fold}}$ (h^−1^)	1.41 [1.30; 1.51] × 10^−3^
CYP2B6 metabolic rate constant, $k_{{\text{met}}_{\text{cyp2b6,sm}}}^{\text{fold}}$ (h^−1^)	0.85 [0.60; 1.11] × 10^−4^

### Impact of different rifampicin concentrations on gene expression and metabolic activity of PXR-regulated genes

**Fig 5:**
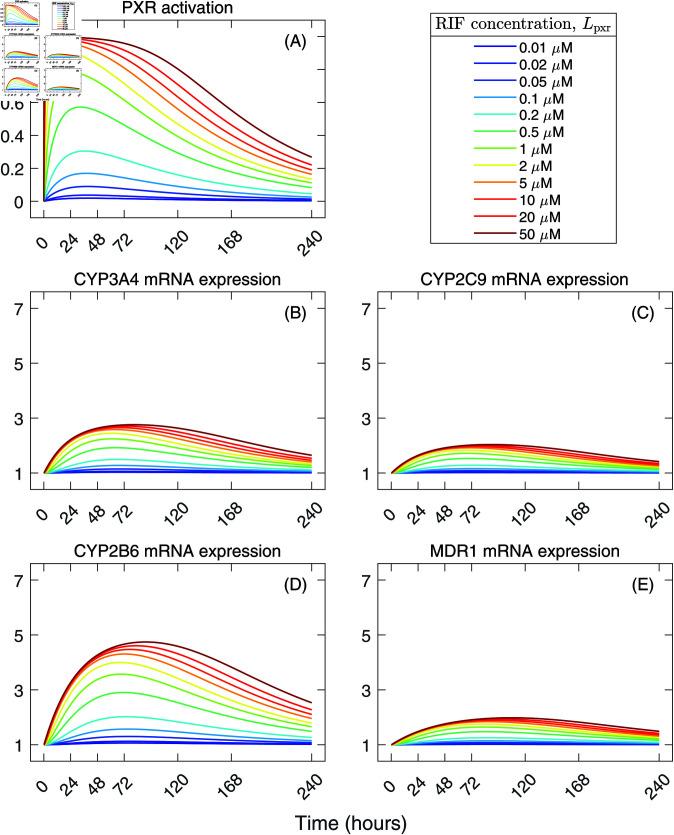
Predicted levels of activated PXR and fold mRNA expression of PXR-regulated genes for different rifampicin concentrations, $L_{\text{pxr}}$. Solutions of the gene expression model (Eq (6)–(10)) were generated using the best-fit parameter values in Table 1. 3D PHHs were assumed to be treated once at time 0 h with rifampicin at indicated concentrations. Rifampicin is assumed not to be metabolized or degraded as demonstrated in [51].

We varied the rifampicin concentration $L_{\text{pxr}}$ to predict changes in the fold mRNA expression of CYP3A4, CYP2C9, CYP2B6, and MDR1 and metabolic activities of CYP3A4, CYP2C9, and CYP2B6. Low concentrations of rifampicin, these were, from 0.01 to 0.1 µM, activated PXR from 2% to 17%, respectively, of its full capacity (Fig 5A). However, such low concentrations did not induce any positive effect on the fold mRNA expression levels (Fig 5B–5E). Rifampicin concentrations of at least 0.2 µM activated PXR at the peak level of at least 31% and were sufficient to induce noticeable changes in the gene expression levels. Rifampicin concentration of 5 µM was able to activate PXR almost fully at the peak level of 98%. Increases in the rifampicin concentration caused PXR activation to reach the maximum at earlier times (Fig H in S1 Text) and PXR remained highly activated at late times (Fig 5A).

Gene expression maxima varied with varying rifampicin concentration and were reached at different times (Fig H in S1 Text).

To validate predictions of the gene expression model with respect to varying rifampicin concentrations, we treated 3D PHHs rescued from a donor (further referred to as donor 4) different from donors used to generate the original dataset in Fig 1 (detailed information about donor 4 is in Materials and Methods) and measured CYP3A4 fold mRNA levels at 24 h and 72 h following treatment with rifampicin at concentrations 0.1 µM, 1 µM, 10 µM, and 30 µM (Fig I in S1 Text (panel A)). Gene expression model predicted CYP3A4 fold mRNA levels for donor 4 accurately for the indicated rifampicin concentrations with the exception of the 30 µM measurement at 24 h at which point the measured CYP3A4 fold mRNA levels were 3.0 compared to the predicted 2.21. Notably, CYP3A4 mRNA fold datapoints corresponding to treatment with 1 µM and 10 µM rifampicin fell within prediction bands (Fig I in S1 Text (panels B and C)).

Fig 4J depicts how varying rifampicin concentration alters predictions in the accumulated amounts of 1-OH-midazolam following treatment with a single substrate, midazolam. Fig D in S1 Text (panel J), Fig E in S1 Text (panel J), and Fig F in S1 Text (panel J) depict how varying rifampicin concentration alters predictions in the accumulated amounts of 1-OH-midazolam, 4-OH-diclofenac, and OH-bupropion following treatment with the substrate mixture of midazolam, diclofenac, and bupropion. Metabolic activity of CYP3A4 appeared to be the most sensitive to changes in rifampicin concentrations of all three enzymes as demonstrated by the largest differences in the accumulated metabolite over the course of 240 h.

## Discussion

Ever since the discovery of PXR as a xenobiotic sensor, it has been extensively studied as an essential factor in processes involved in drug toxicity, drug efficacy, and drug-drug interactions (DDIs) [3,4,46,47]. Quantification of the effects of a ligand on the PXR activity is usually conveyed as a change in the mRNA expression of the PXR-regulated genes, the most studied of which have been the members of the cytochrome P450 family (CYPs), those are, the drug-metabolizing enzymes [14,48]. To understand the effects of PXR on the PXR-regulated genes, it is pivotal to evaluate the short- and long-term effects of PXR activation on gene expression *in vitro*. Experimental *in vitro* treatment studies of traditionally cultured monolayers of PHHs have been limited to only the short-term effects of PXR activation because PHHs exhibit significant de-differentiation shortly after seeding. Consequently, the mathematical models of the PXR-mediated CYP induction *in vitro* have been able to capture either only the initial phase of the CYP kinetic profiles [36,37] or their predictions were extrapolated from the dose-response data [38,40], rendering these quantitative models unusable to predict the long-term effects of PXR activation on its target genes. Using the state-of-the-art *in vitro* model of 3D PHH spheroids, our analysis provides a comprehensive description of the short- and long-term effects of PXR activation on the collective mRNA expression dynamics of three important drug-handling enzymes, CYP3A4, CYP2C9, and CYP2B6, including their metabolic activities and one drug transporter, MDR1, under two clinically relevant rifampicin concentrations.

We highlight the consistency between the fits of our gene expression kinetic model and the experimental data. The mRNA fold expression levels of the PXR-regulated genes peaked between 48 h and 72 h post treatment after which they began to decline. In the previous mathematical models of PXR activation, the decline in the postpeak CYP3A4 mRNA fold expression was explained by the negative feedback inhibition of PXR which was dependent on the levels of CYP3A4 protein [36]. However, such a mechanism of action poses a challenge when multiple PXR-regulated genes are considered concurrently as it is unclear whether this negative feedback inhibition of PXR should be primarily driven by CYP3A4 or whether other PXR-regulated genes should participate in the attenuation of PXR activity. Another theoretical study proposed a mechanism whereby CYP3A4 enzyme directly metabolizes rifampicin [37]. However, this proposed mechanism has little support in the literature as the primary metabolite of rifampicin, 25-desacetyl rifampicin, arises via deacetylation facilitated by arylacetamide deacetylase [49,50]. In our recent study [51], we showed that 25-desacetyl rifampicin was detectable in 2D PHHs 48 h following treatment with 10 µM rifampicin but was not detectable in 3D PHHs even at 72 h following treatment with 10 µM rifampicin. Although the lack of detectable 25-desacetyl rifampicin in 3D PHHs could be attributed to the significantly lower cell density in 3D PHHs (1,500 cells) compared to 2D PHH monolayer culture (350,000 cells) [52], these results still suggest that rifampicin metabolism might not be the primary driver of PXR loss of action in 3D PHHs. The decline in the postpeak CYP3A4 mRNA expression could also be attributed to a rapid and spontaneous rifampicin degradation in the PHH medium. To elucidate the extent to which rifampicin remains stable in the PHH medium over time, we measured the concentration of rifampicin in the PHH medium and in the PHH medium containing 3D PHHs lysate at 24 h and 72 h post rifampicin treatment [51]. The concentrations of rifampicin were comparable with 7.9 ± 0.4 µM in the PHH medium and 9.1 ± 1.7 µM in the PHH medium containing 3D PHH lysate at 24 h and 7.0 ± 0.6 µM in the PHH medium and 8.7 ± 0.7 µM in the PHH medium containing 3D PHH lysate at 72 h. These slight decreases in the rifampicin concentration between 24 h and 72 h were compensated by the rapid transformation of rifampicin into rifampicin quinone which exhibits comparable affinity to and ability to activate PXR as rifampicin. Since accumulated concentrations of rifampicin and rifampicin quinone were comparable to the initial rifampicin concentration of 10 µM, it is reasonable to assume that the total concentration of rifampicin and its derivates does not change over time and the assumption of the constant ligand concentration $L_{\text{pxr}}$ in the gene expression mathematical model is justifiable.

To resolve the aforementioned conflicts in the literature, we introduced a mechanism independent of the mRNA and protein levels of CYP3A4 and assumed that the rate constant controling PXR activation was time-dependent and reduced in time. Such a reduction in PXR activation rate could be possible due to (a) exhaustion of other transcription factors and their cofactors required for successful initiation of the transcription process or (b) the cross-talk with the cell-signalling pathways which could result in PXR phosphorylation [53]. Without the time-dependent PXR activation rate constant, that is, $k_{\text{r}}=0$, the activated PXR settles at the positive equilibrium $k_{\text{pxr,max}}∕(k_{\text{pxr,max}}\;L_{\text{pxr}}\;+\;k_{\text{pxr,deg}})$ and consequently, the mRNA fold expression levels settle at the equilibria that are above the baseline levels, that is, above 1 (Fig J in S1 Text), which is in conflict with our experimental results. Moreover, the gene expression model with the time-dependent PXR activation rate constant, that is, when $k_{\text{r}}>0$, yielded significantly better fits (measured by the Bayesian Information Criterion) than the gene expression model in which the time-dependent PXR activation rate constant was not considered (Fig K in S1 Text).

Fitting the gene expression model to the gene mRNA fold expression data produced parameter estimates, of which only the PXR-dependent CYP mRNA fold transcription and CYP mRNA degradation rate constants were directly comparable to the values presented in the existing literature. Specifically, in [36] the PXR-dependent CYP3A4 mRNA fold transcription rate, $k_{{mRNA}_{\text{cyp3a4}}}^{\text{fold}}$, was estimated to be almost fifteen times higher (1.224 h^−1^, calculated as the multiplication of the parameter *q* and the CYP3A4 mRNA degradation rate $k_{\text{rna,deg}}$) than the value we estimated in our analysis (0.083 h^−1^). However, such a difference could be attributed to the usage of de-differentiated 2D PHH monolayer assay in [36]. The estimate of the CYP3A4 mRNA degradation rate constant, $k_{{mRNA}_{\text{cyp3a4,deg}}}$, that resulted from our analysis (0.044 h^−1^) was comparable to that reported in [36] (0.028 h^−1^) and in [9] (0.038–0.05 h^−1^), but was lower than those reported in [39] (0.23 h^−1^ for CYP3A1 mRNA and 0.197 h^−1^ for CYP3A2 mRNA) and in [37] (2.4 h^−1^). These differences in the CYP3A mRNA degradation rate constants could stem from the differences in the estimation approaches or the fact that in our study, we estimated the CYP3A4 mRNA degradation rate constants directly from the CYP3A4 mRNA degradation data whereas in these other theoretical studies, this parameter was estimated from the CYP3A mRNA time course expression kinetic profiles.

Exposure of 3D PHHs to the PXR antagonist SPA70 did not result in any down-regulation of MDR1 mRNA (Fig A in S1 Text). It was shown that PXR is functionally relevant in the induction of MDR1 expression, but other nuclear receptors, such as the constitutive androstane receptor (CAR), could play a dominant role at maintaining basal expression of MDR1 [54,55]. Therefore, the MDR1 mRNA degradation rate constant in our gene expression model, $k_{{mRNA}_{\text{mdr1,deg}}}$, was related to and estimated directly from the MDR1 mRNA gene expression data.

We quantified the CYP-specific metabolic rate constants and predicted the levels of accumulated CYP-specific metabolites over time, which differed substantially between CYPs. Moreover, we compared the metabolic action of CYP3A4 in 3D PHHs following treatment with the CYP3A4-specific probe substrate, midazolam, alone (single substrate) and with a mixture of CYP-specific substrates, midazolam (CYP3A4), diclofenac (CYP2C9), and bupropion (CYP2B6) (substrate mixture). CYP3A4 metabolized midazolam at a lower rate in the single substrate experiment than in the substrate mixture experiment (Table 2 and Fig G in S1 Text). Although the CYP3A4 metabolic rate constant was statistically significantly lower in the single substrate experiment than in the substrate mixture experiment, the posterior histograms of both rate constants partially overlapped. These differences could simply be the result of the variability in the measurements arising from the quantification process. The metabolic rate constant as well as the predicted levels of accumulated CYP-specific metabolite were the highest for CYP3A4 despite that CYP2B6 exhibited the highest mRNA fold expression levels. CYP2B6, in particular, was not efficient in metabolizing bupropion as its metabolic rate constant was more than one order of magnitude lower than those of CYP3A4 and CYP2C9. Such an inversion in mRNA fold expression and activity of CYP3A4 and CYP2B6 might imply differential translational efficacy or protein stability. As reported in [43], while CYP3A4 was highly inducible at both the mRNA and protein expression levels, CYP2B6 induction at the mRNA expression level was notably higher than at the protein expression level. Another factor that might cause these discrepancies is differential binding affinity of midazolam and bupropion to the active site of CYP3A4 and CYP2B6, respectively. Direct comparisons of the metabolic rate constants estimated in this study and the estimated metabolite formation rates in the existing literature are challenging as the latter is simply calculated as the amount of accumulated metabolite over measured time whereas the former reflects the per-unit velocity at which CYP3A4 (in our study indirectly via CYP3A4 mRNA fold induction) transforms the substrate into the metabolite. We emphasize that our approach to model CYP activity dynamically by linking CYP mRNA fold expression and the CYP-specific substrate amounts allowed an easy integration of the CYP mRNA fold expression and activity kinetics and thus allowed to predict the levels of accumulated metabolite at any given time.

Metabolite formation rates have been measured for different CYPs in human and animal models [39,45,56–58]. In [39], for example, the authors estimated the rates of formation of 6*β*-hydroxytestosterone, a prototypic testosterone reaction of the rat CYP3A1 and CYP3A42, following treatment with dexamethasone, by linearly relating the CYP3A1 and CYP3A2 protein levels to the CYP3A1 and CYP3A2 activity measurements. In our study, we related the CYP activity measurements to the CYP-specific mRNA fold expression levels. Such differences in the modelling approaches lead to the changes in the units of metabolite formation rates and rate constants and thus made direct comparisons difficult. In another study [45], the metabolite formation rates for CYP3A4, CYP2C9, and CYP2B6 were quantified in 3D PHHs, however, different probe substrates (to those we used in this study) were used for determining CYP2C9 and CYP2B6 activities. Moreover, associations between mRNA expression, protein level, and enzymatic activity are not always straightforward. Although a positive correlation between mRNA levels and metabolic activity has been reported for CYP3A4 and CYP2B6 in the human liver [59–61], little evidence exists of any significant correlation between CYP2C9 mRNA levels and its metabolic activity either in the human liver [61] or in the human small intestine [62]. Our approach could thus be limited by the lack of clear relationships between CYP2C9 mRNA levels and metabolic activity which could be resolved by incorporating more diverse data, e.g, protein levels, and including the protein interactions in the gene expression and metabolic activity models. We also note that CYP3A5 has been shown to be inducible and to significantly contribute to midazolam hydroxylase activity [63]. Since the contribution of CYP3A5 to the overall formation of 1-OH-midazolam cannot be excluded, prior genotyping of 3D PHHs for CYP3A4 and CYP3A5 polymorphism is desirable.

Modeling the expression kinetics of the PXR-regulated drug-handling genes may present further challenges as the induction of CYPs and MDR1 are not exclusively controlled by PXR, but are simultaneously governed by both, PXR and CAR [47,53]. In particular, CYP2B6 expression is primarily regulated by CAR [64]. Thus, distinguishing and quantifying the individual contributions of PXR and CAR to the overall expression of their target genes is essential to better understand their mechanisms of action. The resolution of the gene expression mathematical model could further be improved by considering the 3D PHH spatial structure. The exposure of 3D PHHs to xenobiotics might be highly non-uniform along the penetration gradient from the upper layers to the center of the sphere, thus generating heterogenous response in the individual PHHs. In their modeling study, Leedale et al. [65] showed that the geometry and permeability of 3D PHH affect the spatio-temporal distribution and dynamics of a drug inside the spheroid. However, in our experimental setup, in which 3D PHHs are constantly exposed to stable concentrations of rifampicin, such heterogeneity in rifampicin disposition could possibly occur in the initial phase of 3D PHHs treatment, but would likely be quickly diminished. Other factors at transcriptional, posttranscriptional, and posttranslational levels may also contribute to CYP induction, such as microRNAs, which have been described to regulate mRNA stability, with several microRNAs identified as direct modulators of CYP mRNA [66]. Although including such factors could improve predictive power of our mathematical model, their modes of action have yet to be thoroughly described in 3D PHHs.

In this study, we provided the first comprehensive quantitative analysis of the long-term activation of PXR and the expression kinetics of the PXR-regulated drug-handling genes in 3D PHHs in response to treatment with two distinct, yet clinically relevant, rifampicin concentrations. Nuclear receptor activation is a complex process which we extensively simplified in our mathematical model. However, such a simplification allowed us to describe PXR activation through mRNA expression and metabolic activity data of the PXR-regulated genes with high accuracy which may help optimize and predict the response of 3D PPHs to therapeutic drugs during drug discovery, toxicity testing, and dose optimization.

## Materials and methods

### Chemicals

Rifampicin (a ligand of human PXR), dimethyl sulfoxide (DMSO), bupropion (probe substrate of CYP2B6, B102), OH-bupropion (H3167), diclofenac (probe substrate of CYP2C9, SML3086), 4-OH-diclofenac (32412), SPA70 (human PXR antagonist) were all gained from Sigma-Aldrich (USA, now part of Merck). Midazolam (probe substrate of CYP3A4, M343000) and its 1-OH derivative (H948420) were acquired from Toronto Research Chemicals (Canada). Oxazepam, carbamazepine, and ketoprofen were received from Merck KGaA (Germany). Acetic acid ( ≤ 99%) was obtained from Honeywell Research Chemicals (Germany). All other organic solvents were of LC-MS grade and purchased from Merck KGaA (Germany) or VWR (Czech Republic). Milli-Q water was produced by a Millipore purification system (MerckMillipore, Germany). Stock solutions were prepared in DMSO.

### Cell culture

Primary human hepatocytes (PHHs) were seeded onto ultra-low attachment 96-well plates (Corning, New York, USA) at a density of 1500 cells/100 µl/well to generate 3D spheroids as previously described [9,56]. Three different donors were provided by BioIVT (Westbury, New York, USA).

### mRNA degradation

Degradation of CYP3A4 and CYP2C9 mRNA expression were previously determined in our study [9]. To evaluate the degradation of CYP2B6 mRNA expression, 3D PHHs were first exposed to DMSO control or rifampicin (10 µM) for 72 h followed by treatment only with SPA70 (10 µM, PXR antagonist) for another 12, 24, 36 or 48 h. Fold changes of mRNA levels at 12, 24, 36, and 48 h of SPA70 treatment were related to mRNA levels at 72 h of rifampicin treatment (set as 1). Regarding MDR1 mRNA degradation, 3D PHHs were treated with SPA70 (10 µM) or DMSO control for the indicated time ranging from 8–168 h, commenced from the day 8 after seeding. Conditioned serum-free PHH medium was changed every 2–3 days of the treatment. mRNA expression of MDR1 was analyzed by RT-qPCR as previously described in [9] using TaqMan assay (FAM) ABCB1 (Hs00184500_m1) gained from Thermo Fisher Scientific (USA).

### mRNA expression kinetic profiles

The data used are from our previous study Smutny et al. [9]. Briefly, 3D PHHs from three different donors were treated with rifampicin at the concentrations of 1 µM (low concentration) and 10 µM (high concentration). Spheroids were harvested at time points 4, 8, 12, 24, 48, 72, 120, 168 h (and in the case of the high concentration treatment newly also at 240 h) and the total RNA was extracted to determine the concentration levels of CYP3A4, CYP2B6, CYP2C9, and MDR1 mRNA. Quantification of mRNA was performed by RT-qPCR as previously described in [9] and the results were expressed as the relative fold change expression to the corresponding vehicle (0.1% DMSO) control at the same time point (set as 1). Each biological replicate was run in technical duplicates. We note that the culture medium enriched with rifampicin was changed at 72 and 120 h. Details of all experiments are in Smutny et al. [9].

### Validation of CYP3A4 fold mRNA predictions by mathematical model

Deep-frozen PHHs derived from a new donor (referred to as Donor 4, male, 58 years old, African American) were treated with rifampicin at the concentrations of 0.1, 1, 10, and 30 µM. Analysis was conducted at the level of CYP3A4 mRNA at two distinct time points, 24 and 72 h post rifampicin treatment. Quantification and presentation of results were performed as described above.

### LC-MS/MS

NexeraX2 UHPLC system coupled to LCMS-8030 triple quadrupole mass spectrometer with electrospray ionization source (ESI) (Shimadzu Corporation, Kyoto, Japan) was employed for LC-MS/MS analysis. The chromatographic column Luna Phenyl-Hexyl (100 × 3 mm, particle size 3 µm) with the guard column containing C18 cartridge (4 × 2 mm, ID) (Phenomenex, USA) was selected based on the previously published method [67]. The mobile phase consisted of 0.05% acetic acid (A) and acetonitrile containing 0.05% acetic acid (v/v) (B). The following gradient elution was used: 0.0-3.3 min (35% B), 3.3-3.4 min (35 → 55% B), 3.4-5.2 min (55% B), 5.2-5.3 min (55 → 95% B), 5.3-7.0 min (95% B), 7.0-7.1 min (95 → 35% B), and 7.1-8.5 min (35% B). The flow rate was 0.4 ml/min, and the column temperature was adjusted to 35*deg* ⁡   C. A sample volume of 5 µl was injected for analysis. Data acquisition and quantification were performed using the LabSolutions software (Version 5.93, Shimadzu, Japan). All analytes were separated. Selected reaction monitoring mode (SRM) was used for analyte quantification. Precursor-to-fragment transitions were monitored for quantification and identification of appropriate analyte. Optimized setting of the MS detector was applied: nebulizing gas flow rate of 2.9 l/min, drying gas flow rate of 20 l/min, DL temperature of 200^∘^C, and heat block temperature of 450^∘^C. Further, the determination of linearity, accuracy, precision, dilution, selectivity, and carry-over is in line with the guidance for Bioanalytical Method Validation, 2018 by the U.S. Food and Drug Administration (available online at: www.fda.gov). The calibration curves were created using the least-square linear regression fit in GraphPad Prism (version 9.3.1., California, USA).

### CYP activity

3D PHHs were treated with rifampicin (10 µM) or DMSO control for 24, 48, 72 or 120 h, commenced from the day 8 after seeding. Conditioned serum-free PHH medium was changed every 2–-3 days of the treatment. Then, 80 µl of medium was aspired from each well and replaced with equal volume of serum-free PHH medium enriched with midazolam (10 µM) alone or along with diclofenac (10 µM) and bupropion (40 µM). 3D PHHs were further incubated for 4 h and medium collected. Produced metabolites 1-OH-midazolam (CYP3A4), 4-OH-diclofenac (CYP2C9), and OH-bupropion (CYP2B6) were analyzed by LC-MS/MS. The amount of the metabolite at 4 h was expressed in pmol per well.

### Mathematical models

#### Gene expression model

The activation of PXR in 3D PHHs is achieved through treatment with the PXR-specific ligand rifampicin at the concentration $L_{\text{pxr}}$. The total rifampicin concentration $L_{\text{pxr}}$ is assumed to be the aggregate of intracellular rifampicin concentration in 3D PHHs and extracellular rifampicin concentration in the cell culture medium. In [51], we demonstrated that rifampicin is maintained at high concentrations over at least 72 h and no detectable amount of rifampicin was metabolized in 3D PHHs. Since rifampicin-enriched PHH medium was refreshed every 48 to 72 h in our experiments and thus kept at steady levels, we assume the rifampicin concentration, $L_{\text{pxr}}$, to be fixed over time. Rifampicin is assumed to be instantaneously transported into 3D PHHs and activate free PXR, $\left({PXR}_{\text{tot}}\;-\;[PXR]\right)$, where [*PXR*] is the amount of activated PXR and ${PXR}_{\text{tot}}$ is the total amount of PXR. We assume that the total amount of PXR available in 3D PHHs is fixed because in 3D PHHs, the PXR mRNA remains at the baseline level over time [9]. We assume that free PXR is activated by rifampicin at the time-dependent rate $k_{\text{pxr}}(t)=k_{\text{pxr,max}}\;e^{-k_{\text{r}}\;t}$, where $k_{\text{pxr,max}}$ is the maximum PXR activation rate constant and $k_{\text{r}}$ is the rate constant controling the time-dependent reduction in PXR activation rate. The time-dependent rate of PXR activation, $k_{\text{pxr}}(t)$, was introduced to reflect the diminishing ability of PXR to initiate transcription of its target genes. The consequences of assuming a constant PXR activation rate are discussed in Discussion and S1 Text. Finally, activated PXR degrades at the rate constant $k_{{\text{pxr}}_{\text{deg}}}$.

In the following description, we use the subscript “gene” to refer to the PXR-regulated genes, that is, CYP3A4, CYP2C9, CYP2B6, and MDR1, respectively, that is gene ∈ { CYP3A4; CYP2C9; CYP2B6; MDR1 } . The PXR-dependent transcription of a gene occurs at the rate constant $k_{{mRNA}_{\text{gene}}}$. In the absence of the PXR ligand, we assume that mRNA is transcribed at the rate constant $p_{{mRNA}_{\text{gene,back}}}$ (herein referred to as the background transcription rate constant). Degradation of mRNA occurs at the rate constant $k_{{mRNA}_{\text{gene,deg}}}$. The full mathematical model of the ligand-induced PXR activation and its action on its target genes is


$$\begin{array}{ccc} \frac{d[PXR]}{dt} & =k_{\text{pxr}}(t)({PXR}_{\text{tot}}-[PXR])\;L_{\text{pxr}}-k_{{\text{pxr}}_{\text{deg}}}\;[PXR], & \end{array}$$ (1)

$$\begin{array}{ccc} \frac{d[{mRNA}_{\text{cyp3a4}}]}{dt} & =k_{{mRNA}_{\text{cyp3a4}}}\;[PXR]+p_{{mRNA}_{\text{cyp3a4,back}}}-k_{{mRNA}_{\text{cyp3a4,deg}}}\;[{mRNA}_{\text{cyp3a4}}], & \end{array}$$ (2)

$$\begin{array}{ccc} \frac{d[{mRNA}_{\text{cyp2c9}}]}{dt} & =k_{{mRNA}_{\text{cyp2c9}}}\;[PXR]+p_{{mRNA}_{\text{cyp2c9,back}}}-k_{{mRNA}_{\text{cyp2c9,deg}}}\;[{mRNA}_{\text{cyp2c9}}], & \end{array}$$ (3)

$$\begin{array}{ccc} \frac{d[{mRNA}_{\text{cyp2b6}}]}{dt} & =k_{{mRNA}_{\text{cyp2b6}}}\;[PXR]+p_{{mRNA}_{\text{cyp2b6,back}}}-k_{{mRNA}_{\text{cyp2b6,deg}}}\;[{mRNA}_{\text{cyp2b6}}], & \end{array}$$ (4)

$$\begin{array}{ccc} \frac{d[{mRNA}_{\text{mdr1}}]}{dt} & =k_{{mRNA}_{\text{mdr1}}}\;[PXR]+p_{{mRNA}_{\text{mdr1,back}}}-k_{{mRNA}_{\text{mdr1,deg}}}\;[{mRNA}_{\text{mdr1}}]. & \end{array}$$ (5)

We provide our experimental gene expression data as the relative change in the mRNA expression level at the time point in the presence of rifampicin to the corresponding level at the same time point in the absence of rifampicin. In the absence of rifampicin, the mRNA levels are assumed to be at their rifampicin-free steady states, ${mRNA}_{\text{gene}}^{*}=p_{{mRNA}_{\text{gene,back}}}∕k_{{mRNA}_{\text{gene, deg}}}$. Therefore, if we divide Equation (1) by $PXR_{\text{tot}}$ (that is, $[pxr]=[PXR]∕{PXR}_{\text{tot}}$) and Equations (2)–(5) by their respective rifampicin-free steady states $mRNA_{\text{gene}}^{*}$ (that is, $[{mRNA}_{\text{gene}}^{\text{fold}}]=[{mRNA}_{\text{gene}}]∕{mRNA}_{\text{gene}}^{*}$), we obtain the system


$$\begin{array}{ccc} \frac{d[pxr]}{dt} & =k_{\text{pxr}}(t)(1-[pxr])\;L_{\text{pxr}}-k_{{\text{pxr}}_{\text{deg}}}\;[pxr], & \end{array}$$ (6)

$$\begin{array}{ccc} \frac{d[{mRNA}_{\text{cyp3a4}}^{\text{fold}}]}{dt} & =k_{{mRNA}_{\text{cyp3a4}}^{\text{fold}}}\;[pxr]+k_{{mRNA}_{\text{cyp3a4,deg}}}\;(1-[{mRNA}_{\text{cyp3a4}}^{\text{fold}}]), & \end{array}$$ (7)

$$\begin{array}{ccc} \frac{d[{mRNA}_{\text{cyp2c9}}^{\text{fold}}]}{dt} & =k_{{mRNA}_{\text{cyp2c9}}^{\text{fold}}}\;[pxr]+k_{{mRNA}_{\text{cyp2c9,deg}}}\;(1-[{mRNA}_{\text{cyp2c9}}^{\text{fold}}]), & \end{array}$$ (8)

$$\begin{array}{ccc} \frac{d[{mRNA}_{\text{cyp2b6}}^{\text{fold}}]}{dt} & =k_{{mRNA}_{\text{cyp2b6}}^{\text{fold}}}\;[pxr]+k_{{mRNA}_{\text{cyp2b6,deg}}}\;(1-[{mRNA}_{\text{cyp2b6}}^{\text{fold}}]), & \end{array}$$ (9)

$$\begin{array}{ccc} \frac{d[{mRNA}_{\text{mdr1}}^{\text{fold}}]}{dt} & =k_{{mRNA}_{\text{mdr1}}^{\text{fold}}}\;[pxr]+k_{{mRNA}_{\text{mdr1,deg}}}\;(1-[{mRNA}_{\text{mdr1}}^{\text{fold}}]). & \end{array}$$ (10)

Such a transformation reduces the number of model parameters from sixteen to eleven, where $k_{{mRNA}_{\text{gene}}^{\text{fold}}}=k_{{mRNA}_{\text{gene}}}\;{PXR}_{\text{tot}}∕{mRNA}_{\text{gene}}^{*}$. Description of the parameters from Equations (6)–(10) are given in Table 1.

#### Metabolic activity model

The metabolite quantities are expressed as the amount of substance (pmol) calculated as the multiplication of the metabolite concentration (µM) and the medium volume (80 µl). Metabolic activities of CYP3A4 and CYP2B6 enzymes have been shown to positively correlate with their respective mRNA levels in the human liver [59–61], suggesting that regulation of CYP3A4 and CYP2B6, specifically, is pre-translational and their mRNA levels could allow for reasonable activity estimations. Thus, we assume that metabolism of the CYP-specific substrate $\left[S_{\text{cyp}}\right]$ is proportional to the CYP mRNA levels and the CYP-specific metabolite $\left[M_{\text{cyp}}\right]$ is produced at the rate constant $k_{{\text{met}}_{\text{cyp}}}$. We assume that the substrate and metabolite degradation over the course of the experiment is negligible and thus omitted. The time course turnover of the substrate into the metabolite is described as


$$\begin{array}{ccc} \frac{d[S_{\text{cyp}}]}{dt} & =-k_{{\text{met}}_{\text{cyp}}}\;[S_{\text{cyp}}]\;[{mRNA}_{\text{cyp}}], & \end{array}$$ (11)

$$\begin{array}{ccc} \frac{d[M_{\text{cyp}}]}{dt} & =k_{{\text{met}}_{\text{cyp}}}\;[S_{\text{cyp}}]\;[{mRNA}_{\text{cyp}}]. & \end{array}$$ (12)

We note that the metabolite formation was experimentally measured only in the cell culture medium. Thus, the rate constant $k_{{\text{met}}_{\text{cyp}}}$ describes the collective contribution of both the intracellular synthesis of the metabolite and its transport out of the cell into the cell culture medium. We considered the metabolic activities of CYP3A4 following treatment with the single substrate (ss), that is, cyp = { cyp3a4,ss } , and of CYP3A4, CYP2C9, and CYP2B6 following treatment with the substrate mixture (sm), that is, cyp ∈ { cyp3a4,sm ; cyp2c9,sm ; cyp2b6,sm } , respectively.

Equations (11)–(12) can be expressed as


$$\begin{array}{ccc} \frac{d[S_{\text{cyp}}]}{dt} & =-k_{{\text{met}}_{\text{cyp}}}^{\text{fold}}\;[S_{\text{cyp}}]\;[{mRNA}_{\text{cyp}}^{\text{fold}}], & \end{array}$$ (13)

$$\begin{array}{ccc} \frac{d[M_{\text{cyp}}]}{dt} & =k_{{\text{met}}_{\text{cyp}}}^{\text{fold}}\;[S_{\text{cyp}}]\;[{mRNA}_{\text{cyp}}^{\text{fold}}], & \end{array}$$ (14)

where $k_{{\text{met}}_{\text{cyp}}}^{\text{fold}}=k_{{\text{met}}_{\text{cyp}}}\;{mRNA}_{\text{cyp}}^{*}$. We recall that 3D PHHs were treated with rifampicin and cultured up to 24 h, 48 h, 72 h, and 120 h. At these times, the medium containing rifampicin was extracted. Let us denote the time at which the medium containing rifampicin was extracted as *T* (that is, *T* ∈ { 24h , 48h , 72h , 120h } ). Following the medium extraction, 3D PHHs were treated with the CYP-specific probe substrates and cultured for 4 h at which point the concentrations of the metabolites were measured. Because the CYP activity experiments were conducted within a short time span (4 hours), during which no significant changes in the CYP mRNA levels were anticipated, we assume that the CYP-associated activity is proportional to the CYP-associated fold mRNA levels at the extraction time *T*, ${mRNA}_{\text{cyp}}^{\text{fold}}(T)$. Thus, the system (13)-(14) can be written as


$$\begin{array}{ccc} \frac{d[S_{\text{cyp}}]}{dt} & =-k_{{\text{met}}_{\text{cyp}}}^{\text{fold}}\;[S_{\text{cyp}}]\;{mRNA}_{\text{cyp}}^{\text{fold}}(T), & \end{array}$$ (15)

$$\begin{array}{ccc} \frac{d[M_{\text{cyp}}]}{dt} & =k_{{\text{met}}_{\text{cyp}}}^{\text{fold}}\;[S_{\text{cyp}}]\;{mRNA}_{\text{cyp}}^{\text{fold}}(T). & \end{array}$$ (16)

By solving the system (15)-(16), we obtain the solutions


$$\begin{array}{ccc} \left[S_{\text{cyp}}\right] & =S_{\text{cyp},0}\;e^{-k_{{\text{met}}_{\text{cyp}}}^{\text{fold}}\;{mRNA}_{\text{cyp}}^{\text{fold}}(T)\;t}, & \end{array}$$ (17)

$$\begin{array}{ccc} \left[M_{\text{cyp}}\right] & =S_{\text{cyp},0}\;\left(1-e^{-k_{{\text{met}}_{\text{cyp}}}^{\text{fold}}\;{mRNA}_{\text{cyp}}^{\text{fold}}(T)\;t}\right), & \end{array}$$ (18)

where $S_{\text{cyp},0}$ is the initial amount of the CYP-specific substrate. Description of the parameters from the Equations (13)–(18) are given in Table 2.

### Parameter estimation

#### Gene expression model.

For estimation of the parameters in Equations (6)–(10), $k_{\text{pxr,max}}$, $k_{\text{r}}$, $k_{\text{pxr,deg}}$, $k_{{mRNA}_{\text{cyp3a4}}^{\text{fold}}}$, $k_{{mRNA}_{\text{cyp3a4,deg}}}$, $k_{{mRNA}_{\text{cyp2c9}}^{\text{fold}}}$, $k_{{mRNA}_{\text{cyp2c9,deg}}}$, $k_{{mRNA}_{\text{cyp2b6}}^{\text{fold}}}$, $k_{{mRNA}_{\text{cyp2b6,deg}}}$, $k_{{mRNA}_{\text{mdr1}}^{\text{fold}}}$, and $k_{{mRNA}_{\text{mdr1,deg}}}$, the following experimental data were considered simultaneously: CYP3A4 mRNA degradation data (11 data points: 4 time points for three donors), CYP2C9 mRNA degradation data (11 data points: 4 time points for three donors), CYP2B6 mRNA degradation data (11 data points: 4 time points for three donors), two sets of CYP3A4 mRNA expression kinetic profiles at the concentrations of rifampicin of 1 µM (24 data points: 8 time points for three donors) and 10 µM (25 data points: 9 time points for three donors), two sets of CYP2C9 mRNA expression kinetic profiles at the concentrations of rifampicin of 1 µM (24 data points: 8 time points for three donors) and 10 µM (25 data points: 9 time points for three donors), two sets of CYP2B6 mRNA expression kinetic profiles at the concentrations of rifampicin of 1 µM (24 data points: 8 time points for three donors) and 10 µM (24 data points: 9 time points for three donors) and two sets of MDR1 mRNA expression kinetic profiles at the concentrations of rifampicin of 1 µM (24 data points: 8 time points for three donors) and 10 µM (24 data points: 9 time points for three donors).

Parameters were estimated using slicesample, the Matlab implementation of the Markov chain Monte Carlo (MCMC) algorithm [68]. To evaluate the goodness of the proposed fit, we maximized the logarithm of the Gaussian likelihood function


−12 (ln ⁡ Ldeg(p→)+ ln ⁡ L(p→)), (19)

where


ln ⁡ Ldeg(p→)=∑gene=CYP3A4CYP2C9CYP2B6 {∑k [ (datagenedeg(tk)−mRNAgenedeg,fold(tk,p→)σgenedeg(tk))2+2ln ⁡  (2πσgenedeg(tk))]} (20)

and


ln ⁡ L(p→)=∑gene=CYP3A4CYP2C9CYP2B6MDR1 {∑k [ (datagene(tk)−mRNAgenefold(tk,p→)σgene(tk))2+2ln ⁡  (2πσgene(tk))]}. (21)

In (20) and (21), $\vec{p}$ is the 11-parameter vector. In (20), ${\text{data}}_{\text{gene}}^{\text{deg}}(t_{k})$ refers to the degradation data points (Fig 1A–C) at the measured time $t_{k}$ and ${mRNA}_{\text{gene}}^{\text{deg,fold}}(t_{k},\vec{p})$ refers to the predicted fold mRNA level at the measured time $t_{k}$, the degradation of which we assume to be exponential and described by


$$\begin{array}{ccc} {mRNA}_{\text{gene}}^{\text{deg}}(t_{k},\vec{p})={mRNA}_{\text{gene}}^{\text{deg}}(0)\;e^{-k_{{mRNA}_{\text{gene,deg}}}\;t_{k}}, & & \end{array}$$ (22)

where $k_{{mRNA}_{\text{gene,deg}}}$ is the rate constant of mRNA degradation and ${mRNA}_{\text{gene}}^{\text{deg}}(0)$ is the amount of mRNA at 72 h post rifampicin treatment. The mRNA level at the time point is expressed as the relative change in the rifampicin-pretreated mRNA amount at the time point to the mRNA amount at the 72 h time point, at which PXR was inhibited by the antagonist SPA70 (thus, ${mRNA}_{\text{gene}}^{\text{deg,fold}}(0)=1$). We express the degradation of mRNA as


$$\begin{array}{ccc} {mRNA}_{\text{gene}}^{\text{deg,fold}}(t_{k},\vec{p})=e^{-k_{{mRNA}_{\text{gene,deg}}}\;t_{k}}. & & \end{array}$$ (23)

In (21), ${\text{data}}_{\text{gene}}(t_{k})$ refers to the gene expression data points (Fig 1D–1K) at the measured time $t_{k}$ and ${mRNA}_{\text{gene}}^{\text{fold}}(t_{k},\vec{p})$ refers to the predicted fold mRNA expression of the gene at the measured time $t_{k}$. All parameter priors were assumed to be uniform.

The parameters $\sigma_{\text{gene}}^{\text{deg}}(t_{k})$ and $\sigma_{\text{gene}}(t_{k})$ in (20) and (21), respectively, denote the standard deviations of all donor data at the measured time $t_{k}$. In the case of a missing measurement, we substituted the missing value with the mean of the two remaining measurements at that time. In the case of two missing measurements, we calculated the standard deviation as the mean of all the standard deviations at all measured time points.

#### Metabolic activity model.

For estimation of the CYP-specific metabolic rate constants in the metabolic activity model (Equations (13)–(14)), $k_{{\text{met}}_{\text{cyp3a4,ss}}}$, $k_{{\text{met}}_{\text{cyp3a4,sm}}}$, $k_{{\text{met}}_{\text{cyp2c9,sm}}}$, and $k_{{\text{met}}_{\text{cyp2b6,sm}}}$, the following experimental data were considered: single substrate CYP3A4 activity data (11 data points: 4 time points for three donors), substrate mixture CYP3A4 activity data (8 data points: 4 time points for two donors), substrate mixture CYP2C9 activity data (8 data points: 4 time points for two donors) and substrate mixture CYP2B6 activity data (8 data points: 4 time points for two donors).

To evaluate the goodness of the proposed fit, we maximized the logarithm of the Gaussian likelihood function


−12ln ⁡ Lact(p), (24)

where


ln ⁡ Lact(p)=∑k [ (datacypact(tk)−Mcyp(tk,p)σcypact)2+2ln ⁡  (2πσcypact)]. (25)

In (25), *p* is the 1-parameter vector, ${\text{data}}_{\text{cyp}}^{\text{act}}(t_{k})$ refers to the CYP-specific metabolic activity data points (Fig 4A–D, Fig D in S1 Text (panels A–D), Fig E in S1 Text (panels A–D), and Fig F in S1 Text (panels A–D)) at the measured time $t_{k}$ and $M_{\text{cyp}}(t_{k},p)$ refers to the predicted CYP-specific metabolic activity (Equation (18)) at the measured time $t_{k}$. All parameter priors were assumed to be uniform. The parameter $\sigma_{\text{cyp}}^{\text{act}}$ denotes the average of all standard deviations across all indicated times for all donors.

### Statistical analysis

Wilcoxon rank sum test ranksum in Matlab (equivalent to Mann-Whitney U-test) was used to determine the statistical significance of parameter differences among individual genes with significance established at p  < 0 . 05. To compare models, the Bayesian Information Criteria (BIC) was used. The model with the lowest BIC was considered the best.

## Supporting information

S1 Text Supporting information (PDF)

S1 Dataset Supporting data (XLSX)
