# Supplementary material for: Quantifying expression and metabolic activity of genes regulated by pregnane X receptor in primary human hepatocyte spheroids
Source: PLoS Comput Biol. 2025 Apr 15;21(4):e1012886. doi: 10.1371/journal.pcbi.1012886 (PMC11999711; doi:10.1371/journal.pcbi.1012886)
Supplement: S1 Text — (PDF) [file pcbi.1012886.s001.pdf]

Quantifying expression and metabolic activity of genes regulated by pregnane X receptor in primary human hepatocyte spheroids

Supporting Information

Lukáš Lochman[1], Ellen Tanaka Kahiya[2∞], Bechara Saade[2∞], Tomáš Smutný[3], Jurjen Duintjer Tebbens[2,4], Petr Pávek[3], and Veronika Bernhauerová[2✉]

[1]Department of Pharmaceutical Chemistry and Pharmaceutical Analysis, Faculty of Pharmacy in Hradec Králové, Charles University, Hradec Králové, Czech Republic
[2]Department of Biophysics and Physical Chemistry, Faculty of Pharmacy in Hradec Králové, Charles University, Hradec Králové, Czech Republic
[3]Department of Pharmacology and Toxicology, Faculty of Pharmacy in Hradec Králové, Charles University, Hradec Králové, Czech Republic
[4]Department of Artificial Intelligence, Institute of Computer Science of the Czech Academy of Sciences, Prague, Czech Republic
[✉]Corresponding author: bernhauve@faf.cuni.cz
[∞]Contributed equally to this work.

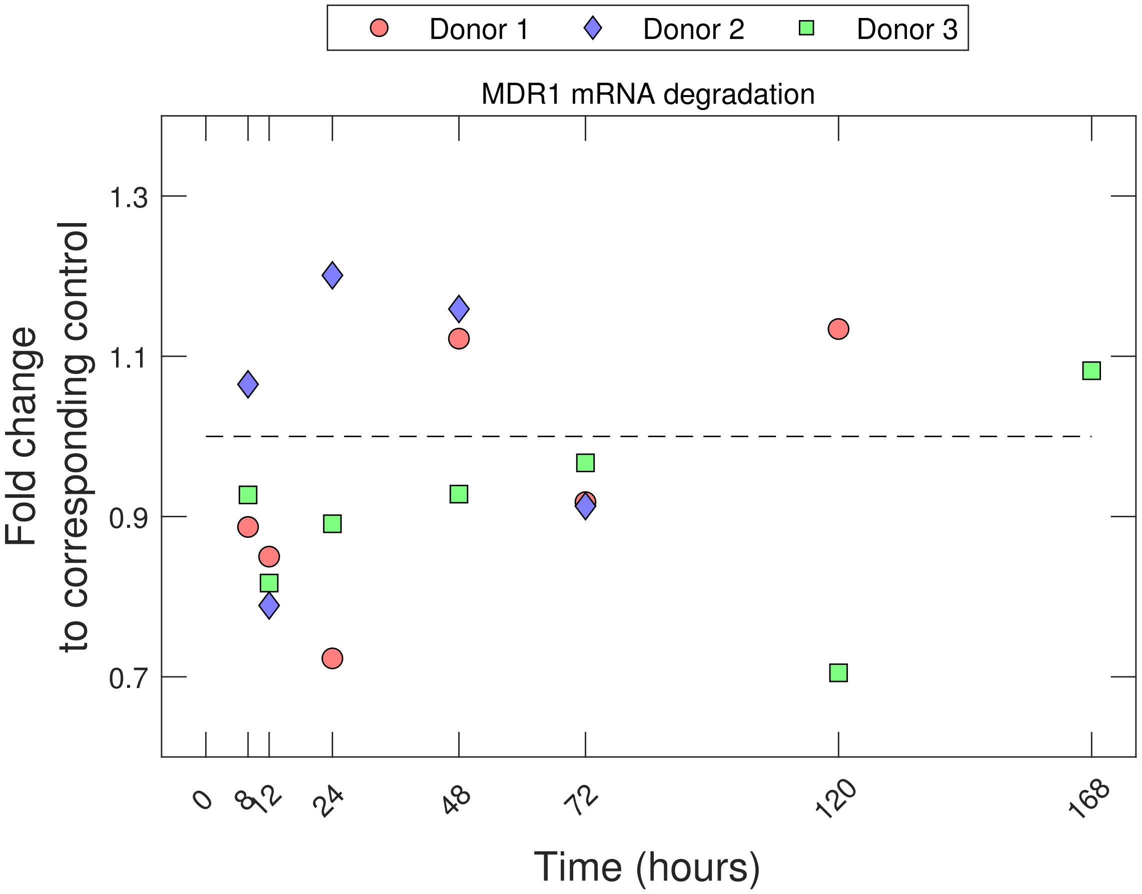

Fig A. MDR1 mRNA degradation. mRNA expression of PXR-controlled MDR1 in 3D PHHs after treatment with SPA70 (10 µM) at indicated time points. Data are shown as the fold change expression relative to corresponding DMSO control (equal to one which is denoted by the broken line).

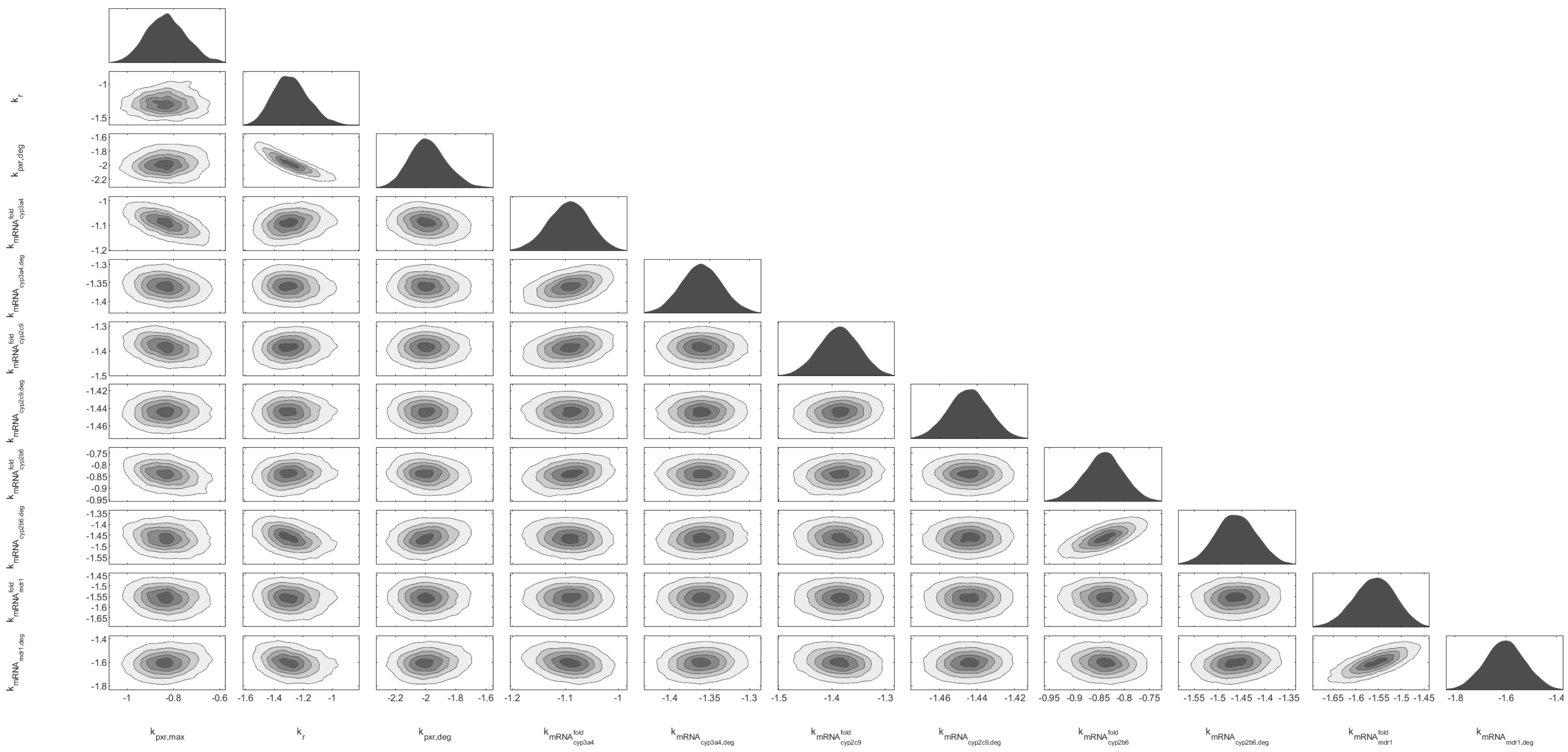

Fig B. Pair-wise parameter posterior distributions. Parameter posterior histograms and pair-wise parameter plots obtained from simultaneous fitting of the gene expression model (Equations (6)–(10) and (23)) to gene expression kinetic data (Fig 2 in the main text) using MCMC. The plot was generated using the function `ecornerplot` from the package `gwmcmc` [1].

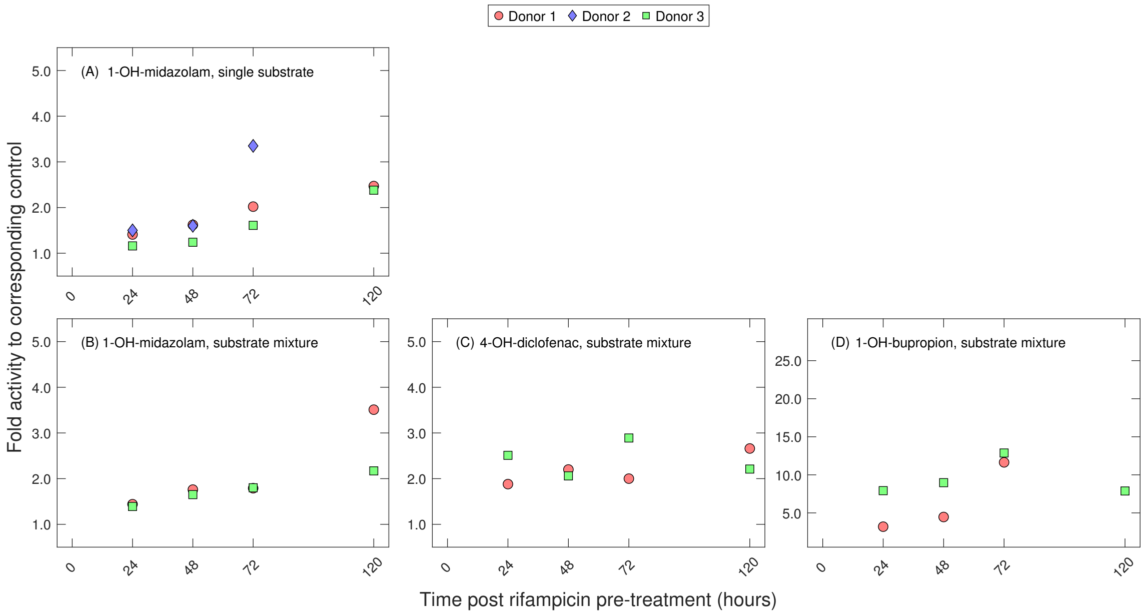

Fig C. CYP3A4, CYP2C9, and CYP2B6 fold activity in 3D PHHs. PHHs were treated with rifampicin (10 µM) or DMSO control for 24 h, 48 h, 72 h, and 120 h. **(A)** In 3D PHHs pre-treated with rifampicin, CYP3A4 activity was evaluated as the formation of 1-OH-midazolam following treatment with midazolam (10 µM) for 4 h. Data were collected from three PHH donors and presented as the fold change activity relative to corresponding DMSO control at the same time point, which was set to be 1. **(B)–(D)** In 3D PHHs pre-treated with rifampicin, CYP3A4, CYP2C9, and CYP2B6 activities were concomitantly evaluated as the formation of 1-OH-midazolam, 4-OH-diclofenac, and OH-bupropion, respectively, following treatment with the mixture of midazolam (10 µM), diclofenac (10 µM), and bupropion (40 µM) for 4 h. Data were collected from two PHH donors and presented as the fold change activity relative to corresponding DMSO control at the same time point, which was set to be 1. We note that at 120 h, in panels (A) and (D), Donor 2 and Donor 1 baseline activities, respectively, were under the limit of detection and therefore are not displayed.

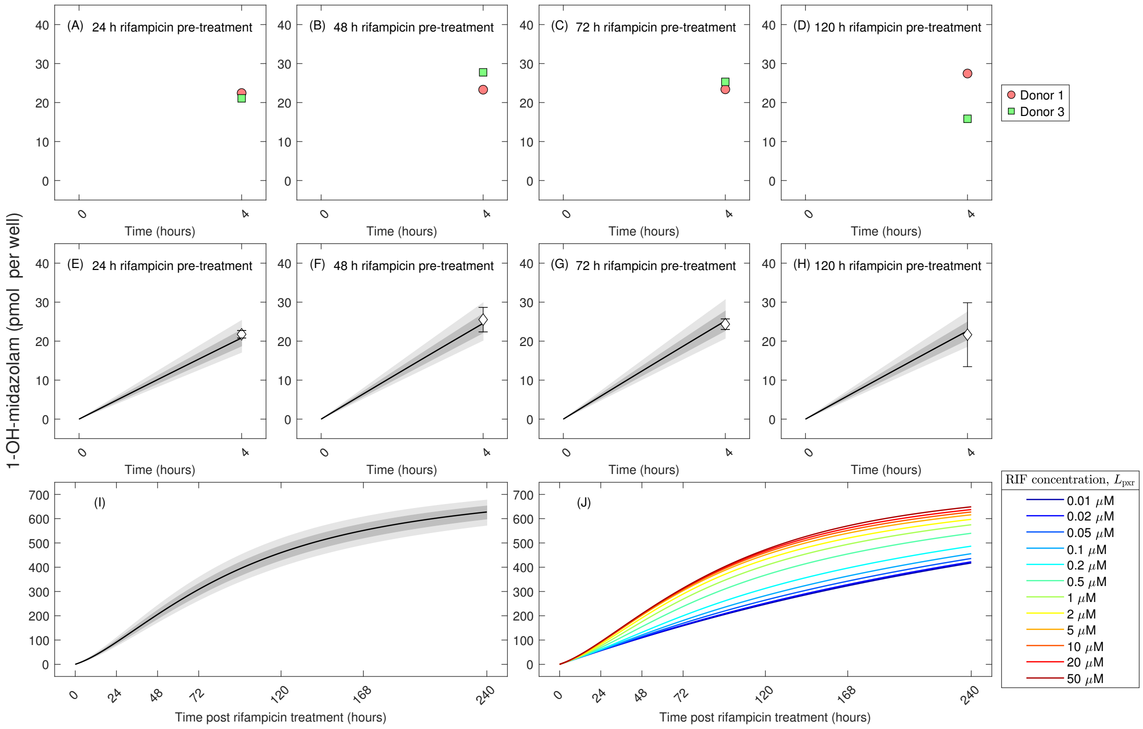

Fig D. CYP3A4 activity in 3D PHHs. **(A)–(D)** 3D PHHs were treated with rifampicin (10 µM) for (A) 24 h, (B) 48 h, (C) 72 h, and (D) 120 h. Then, CYP3A4 activity was evaluated as the formation of 1-OH-midazolam following treatment with the mixture of midazolam (10 µM), diclofenac (10 µM), and bupropion (40 µM) for 4 h. Data were collected from two PHH donors and presented in pmol per well. **(E)–(H)** Fits of Equation (18) in the main text evaluated at CYP3A4 fold mRNA levels at times 24 h, 48 h, 72 h, and 120 h rifampicin post-treatment to 1-OH-midazolam measurements using MCMC. Light filled area represents all MCMC-accepted solutions while dark filled area represents 95% credible bands. Data are displayed as mean ± standard deviation for two donors per time point and are expressed in pmol per well. **(I)** Predicted accumulation of 1-OH-midazolam was generated by solving the gene expression (Equations (6)–(10) in the main text) and metabolic activity (Equations (13)–(14) in the main text) models simultaneously using the best-fit parameter values from Tables 1 and 2 in the main text. The solution corresponding to the MCMC maximum likelihood estimate is displayed as a solid line. Predicted accumulation of 1-OH-midazolam corresponding to the MCMC-accepted values of the parameter $k^{\text{fold}}_{\text{met}_{\text{cyp3a4,sm}}}$ are displayed as the filled area. Light filled area represents all MCMC-accepted solutions while dark filled area represents 95% credible bands. **(J)** Predicted CYP3A4 activities for different rifampicin concentrations, L_{pxr}, were generated using the best-fit parameter values from Tables 1 and 2 in the main text. The initial substrate concentration, $S_{\text{cyp3a4,0}}$, was 800 pmol per well.

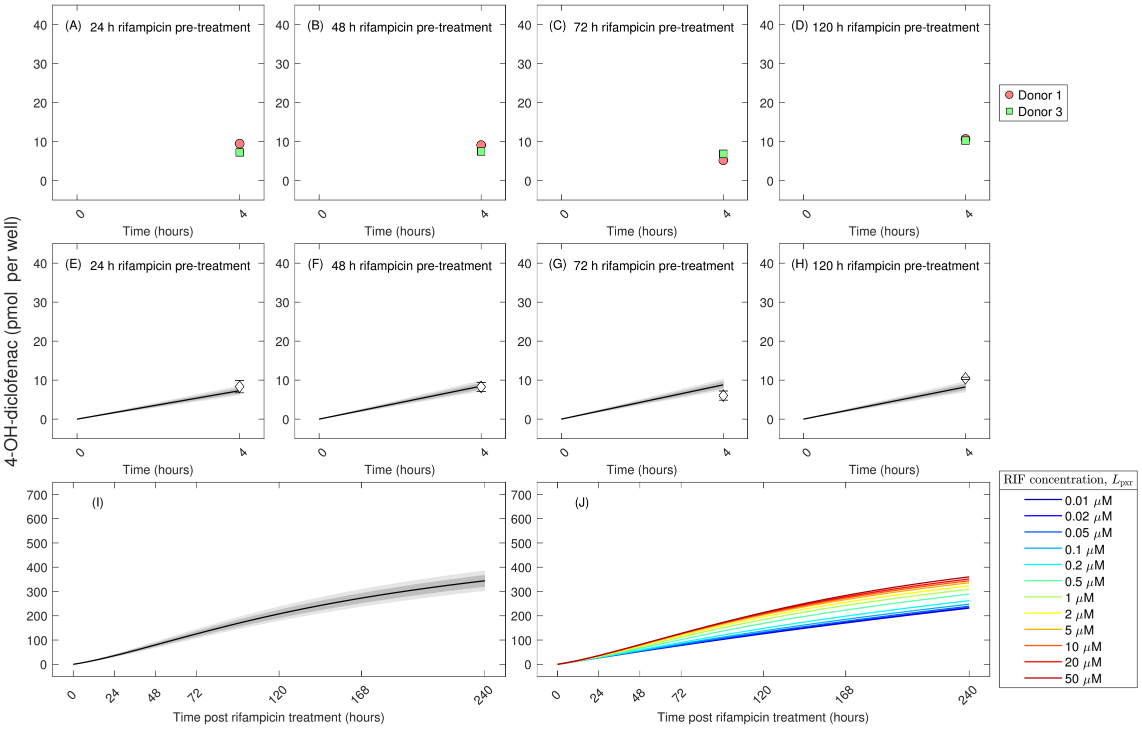

Fig E. CYP2C9 activity in 3D PHHs. **(A)–(D)** 3D PHHs were treated with rifampicin (10 µM) for (A) 24 h, (B) 48 h, (C) 72 h, and (D) 120 h. Then, CYP2C9 activity was evaluated as the formation of 4-OH-diclofenac following treatment with the mixture of midazolam (10 µM), diclofenac (10 µM), and bupropion (40 µM) for 4 h. Data were collected from two PHH donors and presented in pmol per well. **(E)–(H)** Fits of Equation (18) in the main text evaluated at CYP2C9 fold mRNA levels at times 24 h, 48 h, 72 h, and 120 h rifampicin post-treatment to 4-OH-diclofenac measurements using MCMC. Light filled area represents all MCMC-accepted solutions while dark filled area represents 95% credible bands. Data are displayed as mean ± standard deviation for two donors per time point and are expressed in pmol per well. **(I)** Predicted accumulation of 4-OH-diclofenac was generated by solving the gene expression (Equations (6)–(10) in the main text) and metabolic activity (Equations (13)–(14) in the main text) models simultaneously using the best-fit parameter values from Tables 1 and 2 in the main text. The solution corresponding to the MCMC maximum likelihood estimate is displayed as a solid line. Predicted accumulation of 4-OH-diclofenac corresponding to the MCMC-accepted values of the parameter $k^{\text{fold}}_{\text{met}_{\text{cyp2c9,sm}}}$ are displayed as the filled area. Light filled area represents all MCMC-accepted solutions while dark filled area represents 95% credible bands. **(J)** Predicted CYP2C9 activities for different rifampicin concentrations, L_{pxr}, were generated using the best-fit parameter values from Tables 1 and 2 in the main text. The initial substrate concentration, $S_{\text{cyp2c9,0}}$, was 800 pmol/well.

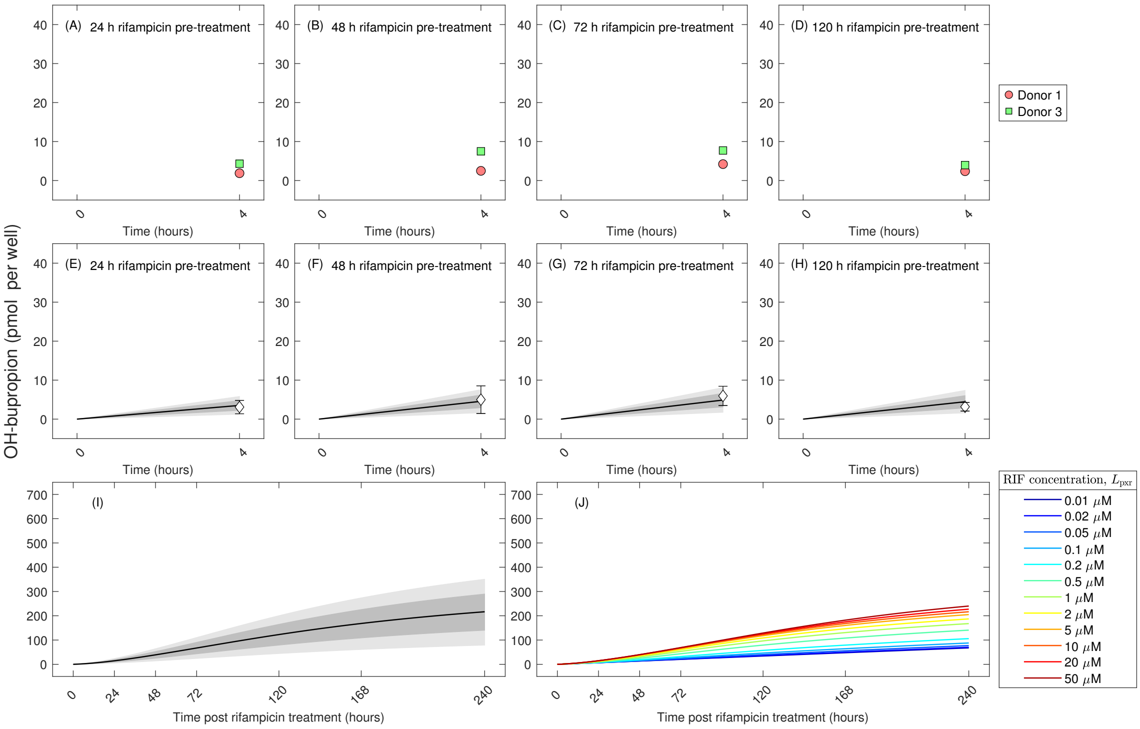

Fig F. CYP2B6 activity in 3D PHHs. **(A)–(D)** 3D PHHs were treated with rifampicin (10 µM) for (A) 24 h, (B) 48 h, (C) 72 h, and (D) 120 h. Then, CYP2B6 activity was evaluated as the formation of OH-bupropion following treatment with the mixture of midazolam (10 µM), diclofenac (10 µM), and bupropion (40 µM) for 4 h. Data were collected from two PHH donors and presented in pmol per well. **(E)–(H)** Fits of Equation (18) in the main text evaluated at CYP2B6 fold mRNA levels at times 24 h, 48 h, 72 h, and 120 h rifampicin post-treatment to OH-bupropion measurements using MCMC. Light filled area represents all MCMC-accepted solutions while dark filled area represents 95% credible bands. Data are displayed as mean ± standard deviation for two donors per time point and are expressed in pmol per well. **(I)** Predicted accumulation of OH-bupropion was generated by solving the gene expression (Equations (6)–(10) in the main text) and metabolic activity (Equations (13)–(14) in the main text) models simultaneously using the best-fit parameter values from Tables 1 and 2 in the main text. The solution corresponding to the MCMC maximum likelihood estimate is displayed as a solid line. Predicted accumulation of OH-bupropion corresponding to the MCMC-accepted values of the parameter $k^{\text{fold}}_{\text{met}_{\text{cyp2b6,sm}}}$ are displayed as the filled area. Light filled area represents all MCMC-accepted solutions while dark filled area represents 95% credible bands. **(J)** Predicted CYP2B6 activities for different rifampicin concentrations, L_{pxr}, were generated using the best-fit parameter values from Tables 1 and 2 in the main text. The initial substrate concentration, $S_{\text{cyp2b6},0}$, was 3200 pmol per well.

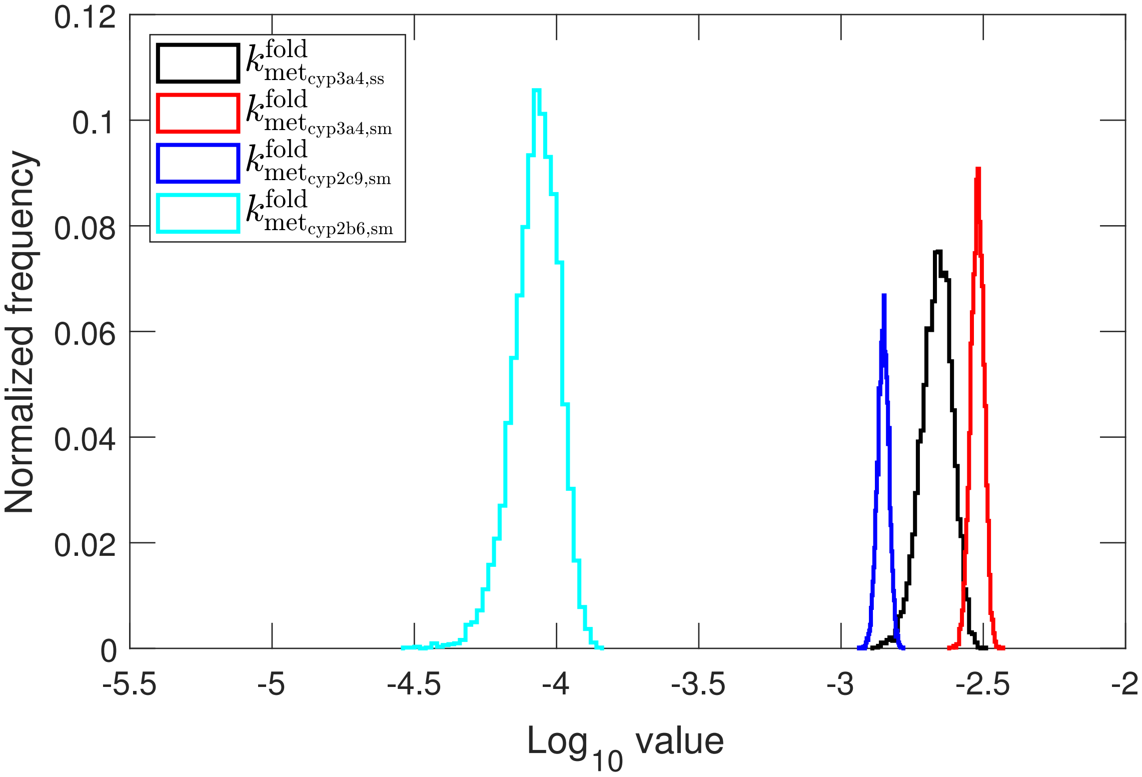

Fig G. CYP-specific metabolic rate constants. Parameter posterior histograms resulting from fitting Equation (18) in the main text to the CYP-specific metabolite measurements at 24 h, 48 h, 72 h, and 120 h rifampicin post-treatment using MCMC (Fig 4 in the main text (panels E–H), Fig D in S1 Text (panels E–H), Fig E in S1 Text (panels E–H), and Fig F in S1 Text (panels E–H)). Parameters shown are metabolic rate constants of CYP3A4 in the single substrate (ss) experiment, $k^{\mathrm{fold}}_{\mathrm{met}_{\mathrm{cyp3a4,sm}}}$, of CYP3A4 in the substrate mixture (sm) experiment, $k^{\mathrm{fold}}_{\mathrm{met}_{\mathrm{cyp3a4\ sm}}}$, of CYP2C9 in the substrate mixture (sm) experiment, $k^{\mathrm{fold}}_{\mathrm{met}_{\mathrm{cyp2c9,sm}}}$, and of CYP2B6 in the substrate mixture (sm) experiment, $k^{\mathrm{fold}}_{\mathrm{met}_{\mathrm{cyp2b6,sm}}}$.

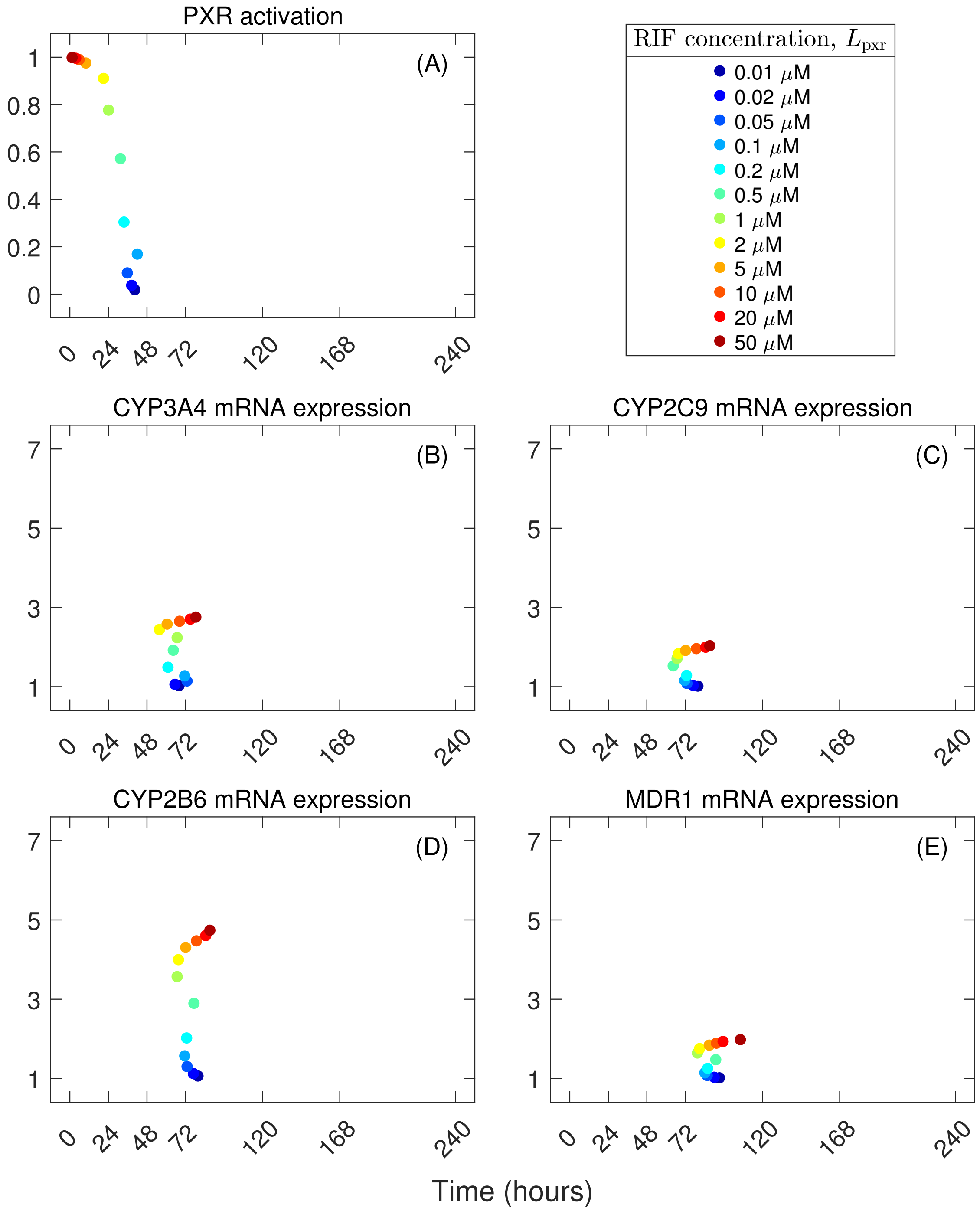

Fig H. Predicted times at which activated PXR and fold mRNA expression levels reach their maxima for different rifampicin concentration, $L_{\mathbf{pxr}}$. Solutions of Equations (6)–(10) in the main text were generated using the best-fit parameter values in Table 1 in the main text.

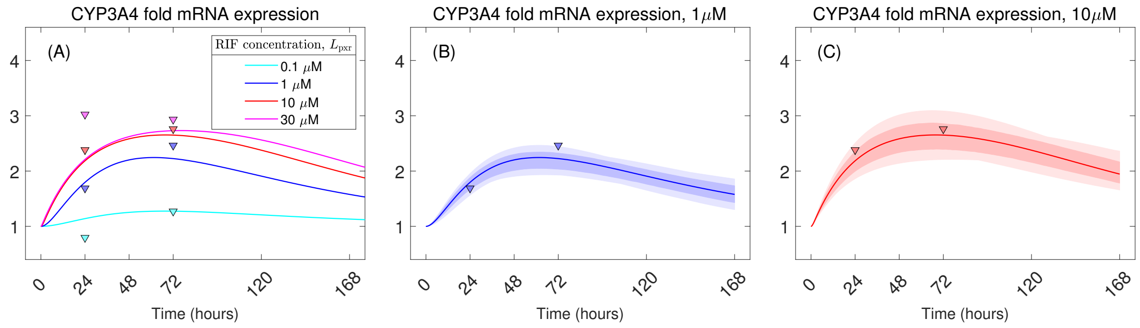

Fig I. Validation of the gene expression model predictions. (A) Predicted levels of CYP3A4 fold mRNA were generated by solving Equations (6)–(7) in the main text at the best-fit parameter values (Table 1 in the main text) and rifampicin concentrations, L_{pxr}, equal to 0.1 µM, 1 µM, 10 µM, and 30 µM. Colored triangles represent CYP3A4 fold mRNA levels at 24 h and 72 h post treatment with rifampicin at concentrations 0.1 µM (cyan), 1 µM (blue), 10 µM (red), and 30 µM (magenta) for Donor 4 (details are in Material and Methods). **(B)–(C)** Fits of the gene expression model for CYP3A4 (Equations (6)–(7) and (23) in the main text) associated with MCMC-accepted parameters (legend as in Fig 2 in the main text) to CYP3A4 mRNA expression data from 3D PHHs treated with (B) 1 µM and (C) 10 µM of rifampicin. Blue (B) and red (C) triangles represent CYP3A4 fold mRNA levels at 24 h and 72 h post treatment with rifampicin at concentrations 1 µM and 10 µM, respectively.

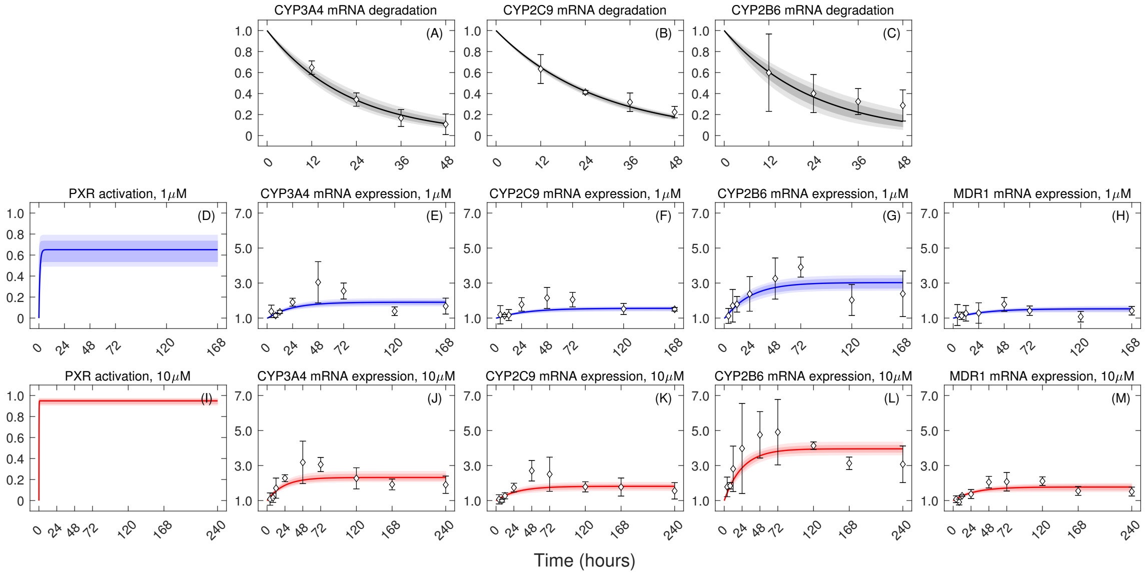

Fig J. Fits of the gene expression model assuming $k_{\mathbf{r}} = 0$ to mRNA expression data. Equations (6)–(10) and (23) in the main text were simultaneously fitted to **(A)–(C)** mRNA degradation data, mRNA expression data from 3D PHHs treated with **(D)–(H)** 1 µM (blue) and **(I)–(M)** 10 µM (red) of rifampicin using MCMC. Accepted parameter sets were used to generate solutions which are displayed as filled areas. Light filled area represents all MCMC-accepted solutions while dark filled area represents 95% credible bands. The solutions corresponding to the MCMC maximum likelihood estimate are displayed as a solid line. Data are displayed as mean ± standard deviation for three donors per time point and are expressed as the fold change expression relative to corresponding DMSO control. The best-fit parameters are in Table A in S1 Text.

Table A. Parameter estimates for gene expression model assuming $k_{\mathbf{r}} = 0$. Best-fit parameter values and 95% credible intervals (CIs) obtained from fitting the gene expression model (Equations (6)–(10) and (23) in the main text) to gene expression and degradation data.

**Parameter**	**Value [95% CI]**
maximum PXR activation rate constant, $k_{\mathrm{pxr,max}}$ (µM^{-1}h^{-1})	0.693 [0.831; 4.240]
activated PXR degradation rate constant, $k_{\mathrm{pxr_{deg}}}$ (h^{-1})	0.371 [0.466; 2.758]
PXR-dependent CYP3A4 mRNA fold transcription rate constant, $k^{\mathrm{fold}}_{mRNA_{\mathrm{cyp3a4}}}$ (h^{-1})	0.063 [0.055; 0.073]
CYP3A4 mRNA degradation rate constant, $k_{mRNA_{\mathrm{cyp3a4,deg}}}$ (h^{-1})	0.045 [0.041; 0.050]
PXR-dependent CYP2C9 mRNA fold transcription rate constant, $k^{\mathrm{fold}}_{mRNA_{\mathrm{cyp2c9}}}$ (h^{-1})	0.031 [0.026; 0.035]
CYP2C9 mRNA degradation rate constant, $k_{mRNA_{\mathrm{cyp2c9,deg}}}$ (h^{-1})	0.036 [0.035; 0.038]
PXR-dependent CYP2B6 mRNA fold transcription rate constant, $k^{\mathrm{fold}}_{mRNA_{\mathrm{cyp2b6}}}$ (h^{-1})	0.130 [0.111; 0.154]
CYP2B6 mRNA degradation rate constant, $k_{mRNA_{\mathrm{cyp2b6,deg}}}$ (h^{-1})	0.042 [0.035; 0.050]
PXR-dependent MDR1 mRNA fold transcription rate constant, $k^{\mathrm{fold}}_{mRNA_{\mathrm{mdr1}}}$ (h^{-1})	0.029 [0.025 0.036]
MDR1 mRNA degradation rate constant, $k_{mRNA_{\mathrm{mdr1,deg}}}$ (h^{-1})	0.035 [0.029; 0.051]

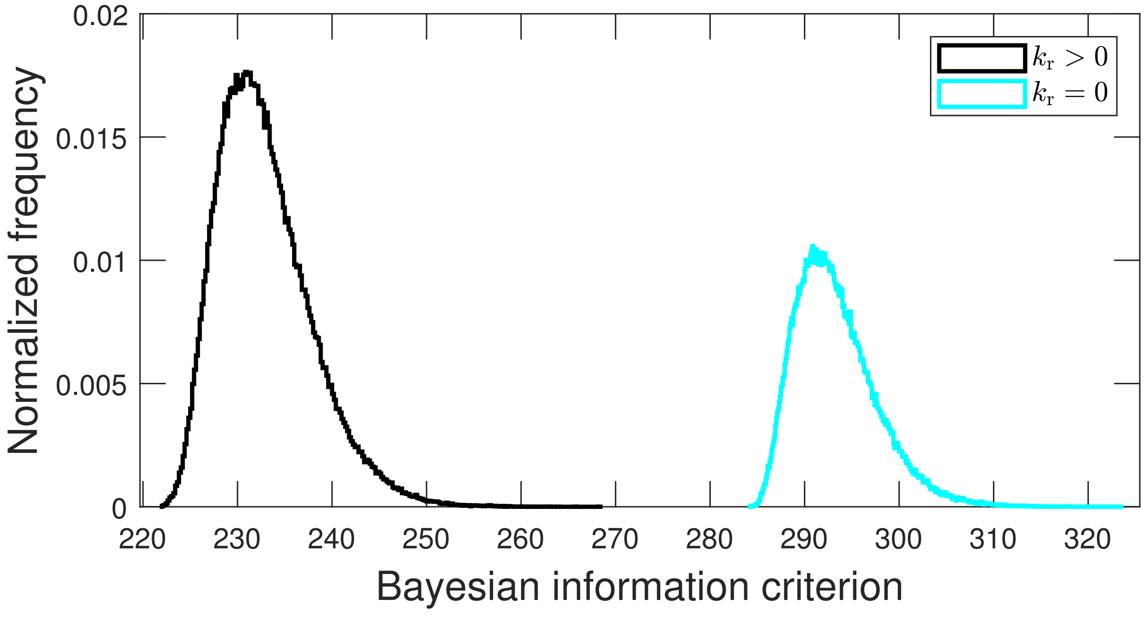

Fig K. Bayesian information criterion for the gene expression model with and without the time-dependent reduction in PXR activation. Bayesian information criterion was constructed using the log-likelihood values that were obtained from fitting the gene expression model (Equations (6)–(10) and Equation (23) in the main text) with $k_r > 0$ and with $k_r = 0$ to the gene expression data (Fig 2 in the main text). Lower values indicate better fits.

References

1. Aslak G. Ensemble MCMC sampler (https://github.com/grinsted/gwmcmc), GitHub. Retrieved December 12, 2024.
